# Suppression of lymphocyte apoptosis in spleen by CXCL13 after porcine circovirus type 2 infection and regulatory mechanism of CXCL13 expression in pigs

**DOI:** 10.1186/s13567-019-0634-2

**Published:** 2019-02-28

**Authors:** Gen Liu, Yanchao Wang, Shijin Jiang, Minmin Sui, Changying Wang, Li Kang, Yi Sun, Yunliang Jiang

**Affiliations:** Shandong Provincial Key Laboratory of Animal Biotechnology and Disease Control and Prevention, College of Animal Science and Veterinary Medicine, Shandong Agricultural University, No. 61 Daizong Street, Tai’an, 271018 Shandong China

## Abstract

Porcine circovirus-associated disease (PCVAD) is one of the most serious infectious diseases in pigs worldwide. The primary causative agent of PCVAD is porcine circovirus type 2 (PCV2), which can cause lymphoid depletion and immunosuppression in pigs. Our previous study demonstrated that Laiwu (LW) pigs, a Chinese indigenous pig breed, have stronger resistance to PCV2 infection than Yorkshire × Landrace (YL) pigs. In this study, we found that the YL pigs showed more severe lymphocyte apoptosis and higher viral load in the spleen tissue than LW pigs. To illustrate the differential gene expression between healthy and infected spleens, transcriptome profiling of spleen tissues from PCV2-infected and control YL pigs was compared by RNA sequencing. A total of 90 differentially expressed genes (DEGs) was identified, including *CD207*, *RSAD2*, *OAS1*, *OAS2*, *MX2*, *ADRB3*, *CXCL13*, *CCR1*, and *ADRA2C*, which were significantly enriched in gene ontology (GO) terms related to the defense response to virus and cell–cell signaling, and another nine DEGs, *KLF11*, *HGF*, *PTGES3*, *MAP3K11*, *XDH*, *CYCS*, *ACTC1*, *HSPH1*, and *RYR2*, which were enriched in GO terms related to regulation of cell proliferation or apoptosis. Among these DEGs, the *CXCL13* gene, which can suppress lymphocyte apoptosis during PCV2 infection, was significantly down-regulated in response to PCV2 infection in YL but not in LW pigs. By analysis of the regulatory elements in the promoter and 3′-untranslated region (3′-UTR) of porcine *CXCL13*, we found that the single nucleotide polymorphism (SNP) −1014 G (LW) > A (YL) and the *Sus scrofa microRNA*-*296*-*5p* (*ssc*-*miR*-*296*-*5p*) participated in regulating *CXCL13* expression during the response to PCV2 infection.

**Electronic supplementary material:**

The online version of this article (10.1186/s13567-019-0634-2) contains supplementary material, which is available to authorized users.

## Introduction

Porcine circovirus type 2 (PCV2) is a virus containing a single-stranded circular DNA genome of only approximately 1.7 kb in size [1]. Currently, PCV2 strains can be separated into five genotypes, composed of three major genotypes, PCV2a, PCV2b, and PCV2d, and two low-prevalence genotypes, PCV2c and PCV2e [2]. Porcine circovirus-associated disease (PCVAD) or porcine circovirus disease (PCVD) caused by PCV2 typically affects weaning piglets of 5–12 weeks and is one of the most important porcine infectious diseases causing enormous economic losses to the swine industry worldwide [3–6].

The hallmark lesions of PCVAD/PCVD occur in lymphoid tissues [3]. PCV2-infected lymphoid tissues show atrophic or necrotizing lesions with deletion of lymphocytes, destruction of lymphoid follicles, and infiltration by large histiocytes and multinucleated giant cells [7]. Lesions are present in the lymph nodes (e.g. superficial inguinal, mesenteric, mediastinal, and submandibular lymph nodes), Peyer’s patches, spleen, thymus, liver, kidney, and lung, resulting in immunosuppression in the pigs [8]. However, the mechanism by which PCV2 causes lymphoid depletion has yet to be identified definitively. Lymphoid depletion can be a direct consequence of viral replication or an indirect consequence of infection, for example due to cell apoptosis. Studies show that PCV2 infection can induce B-lymphocyte apoptosis in vivo and spleen lymphocyte apoptosis in vitro [9, 10].

Genetic differences between individual pigs, strains, or breeds have been demonstrated to play an important role in susceptibility/resistance variation in response to PCV2 infection [11–16]. In 2005, Meerts et al. first reported that the nuclear localization of PCV2 antigens varied significantly between pulmonary alveolar macrophages (PAMs) derived from different individual piglets [13]. Subsequent studies by Opriessnig et al. found that Landrace pigs had more severe clinical symptoms or PCV2-associated microscopic lesions than Duroc, Yorkshire, and Pietrain pigs [14, 15]. Under field conditions, different genetic boar lines could affect the expression of postweaning multisystemic wasting syndrome (PMWS) in their offspring [12]. Our previous study also showed that Laiwu (LW) pigs, a Chinese indigenous pig breed from Shandong province, had less severe symptoms and lower levels of viral load than Yorkshire × Landrace (YL) pigs when they were experimentally challenged with PCV2 strain PCV2-SD, which was isolated from suspected PMWS pigs in Shandong province and belonged to genotype PCV2b [11, 16].

In the present study, we first compared the spleen lesions and viral load at 35 days post-infection (dpi) between LW and YL pigs, and then screened the differentially expressed genes (DEGs) in the spleens of PCV2- and mock-infected YL pigs by RNA sequencing (RNA-seq). The results indicated that LW pigs had less lymphocyte apoptosis and viral load than YL pigs, which might be associated with the different expression pattern of the *CXCL13* (*chemokine CXC ligand 13*) gene between LW and YL pigs after PCV2 infection. Subsequently, we analyzed the regulatory elements in the promoter and 3′-untranslated region (3′-UTR) of porcine *CXCL13* and found a single nucleotide polymorphism (SNP) −1014 G (LW) > A (YL) in the promoter region and *Sus scrofa microRNA*-*296*-*5p* (*ssc*-*miR*-*296*-*5p*) that were likely responsible for the different expression patterns of porcine *CXCL13* gene.

## Materials and methods

### Experimental challenge with PCV2 and sample collection

Fifteen 6-week-old (on average) piglets of purebred LW pigs and crossbred YL pigs, which were both antigen and antibody seronegative for porcine circovirus type 1 (PCV1), PCV2, porcine reproductive and respiratory syndrome virus (PRRSV), and porcine parvovirus (PPV), were selected for experimental challenge as described previously [11]. All pigs were raised under the same conditions and randomly assigned into four groups: PCV2-infected LW pigs (LW-i, *n* = 10), PCV2-uninfected LW pigs (LW-u, *n* = 5), PCV2-infected YL pigs (YL-i, *n* = 10), and PCV2-uninfected YL pigs (YL-u, *n* = 5). The PCV2-SD used in this challenge experiment was isolated from suspected PMWS pigs in Shandong province and belongs to the genotype PCV2b [16]. Pigs from LW-i and YL-i groups were intramuscularly infected with 3 mL PCV2-SD solution (10^3.8^ TCID_50_ (50% tissue culture infective dose)/mL). Pigs from LW-u and YL-u groups were challenged with the same volume of phosphate-buffered saline (PBS). The clinical signs were monitored and recorded every day. Serious clinical symptoms associated with PMWS were observed in PCV2-infected YL pigs, but not in PCV2-infected LW pigs [11]. Each pig in this experiment was sacrificed at 35 dpi. Tissue samples were collected and frozen in liquid nitrogen or fixed by immersion in 10% neutral-buffered formalin.

### Hematoxylin–eosin (HE) and terminal deoxynucleotidyl transferase-mediated dUTP nick-end labeling (TUNEL) staining

Spleen tissues preserved in formalin were embedded in paraffin and sliced at 5 μm thickness. The pathological changes of splenic tissues were observed by HE staining, and were inspected by light microscopy. DNA damage was detected by TUNEL assay using *TransDetect*^®^ In Situ Fluorescein TUNEL Cell Apoptosis Detection Kit following the manufacturer’s protocol (Trans Gen Biotech, Beijing, China), and was visualized by a Fluorescence Inversion Microscope System (NIKON, Japan).

### Quantification of PCV2 DNA

To determine the viral load in the spleens of PCV2-infected LW and YL pigs, the PCV2 genome was extracted from 1 g spleen tissues of these pigs (*n* = 4 per group) with TIANamp Virus DNA/RNA Kit (Tiangen, Beijing, China). The same samples were also used in the following RT-qPCR analysis. The copy numbers of PCV2 DNA in spleen tissues were quantified by absolute quantitative PCR (qPCR) using the following primers: PCV2-F: 5′-GGGCTCCAGTGCTGTTATTC-3′, PCV2-R: 5′-AAGTAGCGGGAGTGGTAGGA-3′) [17] and the nucleotide sequence of them were completely aligned to ORF2 gene of PCV2-SD. A 129 bp was amplified by PCR and cloned into a pMD-18T vector (TaKaRa, Dalian, China). The resultant pMD-18T-PCV2 plasmid was served as a standard DNA template to optimize the assay conditions. The viral load of PCV2 in porcine spleen was analyzed by qPCR with the following conditions: 95 °C for 30 s, 95 °C for 5 s, 55 °C for 30 s and 72 °C for 12 s for 40 cycles. The baseline adjustment method in the Mx3000p software (Stratagene, La Jolla, CA, USA) was used to determine the Ct value. The PCV2 DNA copies of the samples were measured by a linear formula that is established according to the standard curve using the tenfold serial dilutions of the pMD-18T-PCV2 plasmid. All samples were amplified in triplicate.

### RNA-seq

Total RNA was extracted from six spleen samples (samples selected from three mock-infected YL pigs and three PCV2-infected ones) using TRIzol reagent (Invitrogen, Carlsbad, CA, USA), following the manufacturer’s protocol. RNA integrity was evaluated by agarose gel electrophoresis, and RNA concentration and purity were measured by a BioPhotometer plus (Eppendorf, Hamburg, Germany). Total RNA which satisfied the quality for RNA sequencing was delivered to the Biomarker Technologies Corporation (Beijing, China) for cDNA library construction and sequencing. RNA-seq libraries were constructed by NEBNext^®^ mRNA Library Prep Master Mix Set for Illumina and NEBNext^®^ Multiplex Oligos for Illumina (New England Biolabs, Ipswich, MA, USA), and all the procedures and standards were performed according to the protocols for the Illumina HiSeq 2500.

### Analysis of RNA-seq data

The clean reads that were filtered from the raw reads were mapped to the *Sus scrofa* genome (*Sscrofa* 10.2) from the National Center for Biotechnology Information (NCBI) by Tophat software. The gene expression abundance of the six samples was calculated by FPKM (fragments per kilobase of exon per million fragments mapped) values through Cufflinks software [18].

Pairwise correlation was used to estimate the individual variation in the spleen samples. In mock- or PCV2-infected YL pigs, one sample’s R-squared value (R^2^: square of Pearson correlation coefficient) was different from the other two (ranging from 0.8324 to 0.9029; Additional file 1), therefore, was excluded for further DEG analysis. Two samples with high R^2^ values (ranging from 0.9706 to 0.9862) from mock- and PCV2-infected YL pigs were selected for the assessment of the differential expression of mRNA through DESeq [19]. The *P* values were adjusted by the Benjamini–Hochberg method [20], and the false discovery rate (FDR) was obtained. Here, an FDR < 0.05 and absolute value of fold change > 1.5 were set as the threshold for the identification of DEGs.

To determine the functional annotation, all the DEGs were sorted by the enrichment of GO categories in DAVID [21]. The KEGG (Kyoto Encyclopedia of Genes and Genomes) database was used for the pathway enrichment analysis of the DEGs [22].

### Reverse transcription-quantitative PCR (RT-qPCR) analysis

Validation of differentially expressed mRNA and predicted microRNA (miRNA) was performed by RT-qPCR. Spleen tissues from 16 pigs (four groups with *n* = 4 per group), including the six samples used in RNA-seq, were used as substrates for the RT-qPCR, which was conducted on an Mx3000p™ instrument (Stratagene).

To measure the expression level of the mRNA, RNA samples were reverse transcribed to cDNA using the PrimeScript™ RT Reagent Kit with gDNA Eraser (TaKaRa) according to the manufacturer’s instructions. RT-qPCR was performed to detect whether or not a difference in expression patterns of the candidate DEGs existed between LW and YL pigs. Primers for the genes were designed by DNAMAN (version 7.0.2; Lynnon Biosoft, San Ramon, USA). *Sus scrofa* hypoxanthine phosphoribosyl transferase 1 (*HPRT1*) was selected as the endogenous control [23]; all the primer sequences are listed in Additional file 2. The reactions contained 1 μL of cDNA, 7.5 µL of 2 × SYBR Premix Ex Taq (TaKaRa), 0.3 µL of each forward and reverse primer (10 µM), 0.3 µL of 50 × Rox Reference Dye II, and 5.6 µL of sterile distilled water. A twofold dilution series of cDNA (from a pool consisting of 16 samples) was implemented to generate standard curves for each assay and determine the amplification efficiency. Samples were amplified in four biological replicates (nested with three technical replicates) as follows: 1 cycle at 95 °C for 30 s; 40 cycles at 95 °C for 5 s, annealing temperature (Additional file 2) for 30 s, and 72 °C for 20 s. Dissociation curves were performed at the end of amplification to ensure single amplicons.

To measure the expression level of the miRNA, cDNA was reverse transcribed from total RNA using a Mir-X™ miRNA First-Strand Synthesis Kit (TaKaRa), following the manufacturer’s instruction manual, and then used for the quantification of the miRNA. 5S rRNA was used as the internal control as shown in Additional file 2 [16]. The reaction system included 0.6 μL of forward primer (10 μM, Additional file 2), 0.6 μL of mRQ 3′ primer (10 μM; TaKaRa), 10 μL of 2 × SYBR Premix Ex Taq II, 0.4 μL of 50 × ROX reference dye II, 1.5 μL of cDNA, and 6.9 μL of sterile distilled H_2_O. The two-step RT-qPCR program was as follows: 95 °C for 30 s; 40 cycles at 95 °C for 5 s, 60 °C for 40 s; followed by dissociation curves for rising the temperature from 62 to 95 °C.

The 2^−ΔΔCt^ method was used to calculate the relative expression levels of each mRNA and miRNA. Each sample was replicated three times.

### Western blotting

Protein from the sixteen samples (four groups with *n* = 4 per group) used in RT-qPCR was extracted from spleens with Cell Lysis Buffer for Western & IP (Beyotime, Shanghai, China) and used for expression analysis by Western blotting. Protein concentration was measured with a BCA kit (Beyotime). A 75 μg aliquot of each protein sample was loaded and then separated in SDS-PAGE 10% gels (Fdbio science, Hangzhou, China), and transferred to polyvinylidene fluoride membranes (Solarbio, Beijing, China). After blocking for 2 h at room temperature in Western Blocking Buffer (Beyotime), the membranes were incubated overnight at 4 °C with rabbit polyclonal antibody against CXCL13 (1:100 dilution; GenScript, Nanjing, China) or mouse anti-GAPDH monoclonal antibody (1:1000 dilution; Beyotime). The membranes were washed with PBS containing 0.05% Tween-20 (PBST) and incubated with horseradish peroxidase (HRP)-conjugated goat anti-rabbit or anti-mouse IgG (1:1000 dilution; Beyotime) at room temperature for 2 h. The resulting signals were visualized using FDbio-Dura ECL solution A and FDbio-Femto ECL solution B (Fdbio Science), collected by Fusion FX system (Vilber Lourmat, Marne-la-Vallée, France), and measured with ImageJ software [24].

### Flow cytometry

To further clarify the correlation between CXCL13 and lymphopenia, mouse splenocytes were used as a model for exploring the function of the CXCL13 protein. Splenocytes were isolated from spleens of 5- to 8-week-old mice of the Kunming breed using a mouse spleen lymphocyte isolation kit (TBD, Tianjin, China), as described in the manufacturer’s protocol. Splenocytes were seeded at a density of 1 × 10^6^ cells per well into a 12 well cell culture plate (Corning, NY, USA) and infected with PCV2 at a multiplicity of infection (MOI) of 0.1 TCID_50_ in two PCV2-infected groups. As PCV2 was propagated in PK15 cell, the control group was treated with equivalent PK15 cell lysate, and incubated at 37 °C with 5% CO_2_ for 1 h. Subsequently, the medium was replaced by RPMI-1640 (Gibco, Grand Island, NY, USA) with 2% fetal bovine serum (FBS; Biological Industries, Kibbutz Beit Haemek, Israel). Murine CXCL13 protein (Pepro Tech, Rocky Hill, NJ, USA) was added in one of PCV2-treated groups at 1 μg/mL, and the other two groups were treated with RPMI-1640 with 2% FBS (without CXCL13 protein); this time point was regarded as 0 hour post-infection (hpi). The apoptosis rate of the splenocytes was measured by flow cytometer (BD Biosciences, Franklin Lakes, NJ, USA) using a FITC Annexin V Apoptosis Detection Kit (BD) at 2, 4, 8, 16, and 24 hpi.

### Construction of various recombinant plasmids

To determine whether *CXCL13* is regulated at the transcriptional or/and post-transcriptional level, various recombinant plasmids were constructed, covering the 5′- and 3′-regulatory region of *CXCL13*. Firstly, for the study of the 5′-regulatory region of *CXCL13*, primers (CXCL13-F1/R1; Additional file 2) were designed to amplify a 3486 bp DNA fragment according to the genomic sequence of the porcine *CXCL13* gene in the NCBI database, containing 3407 bp of the promoter region and 79 bp of the 5′-untranslated region (5′-UTR) of *CXCL13* from LW and YL pigs. The PCR was conducted in a 25 μL volume containing 8.25 μL of H_2_O, 12.5 μL of 2 × PrimeSTAR GC Buffer (Mg^2+^ Plus), 2 μL of dNTP mixture (2.5 mM), 0.5 μL each of the primers (10 μM), 0.25 μL of PrimeSTAR HS DNA Polymerase (5 U/μL; TaKaRa), and 1 μL of porcine genomic DNA, using the following PCR amplification program: 94 °C for 5 min; then 35 cycles of 98 °C for 10 s, 65 °C for 15 s, and 72 °C for 3.5 min; followed by 72 °C for 7 min. PCR products were purified using a Gel Extraction Kit (CWBIO, Beijing, China), and then digested simultaneously by *Xho*I and *Hin*dIII (Thermo Fisher Scientific, Waltham, MA, USA). The products were purified once more, ligated into the pGL3-Basic vector (Promega, Madison, WI, USA), and sequenced. The recombinant plasmids were named pGL3-CXCL13LW and pGL3-CXCL13YL.

Based on the promoter sequence from YL pigs, variable-length DNA fragments were amplified by different forward primers (CXCL13-F2 to CXCL13-F6; Additional file 2) and CXCL13-R to construct serial deletion promoter reporters, which were named pGL3-CXCL13(−2666/+ 79), pGL3-CXCL13(−2205/+ 79), pGL3-CXCL13(−1532/+ 79), pGL3-CXCL13(−1089/+ 79), and pGL3-CXCL13(−589/+ 79), respectively.

The promoter sequences of *CXCL13* from LW and YL pigs were aligned using DNAMAN software to search for SNPs in the pivotal segment. To assess the effect of these on the transcriptional activity of the promoter, pGL3-CXCL13YL plasmid was used as a template to design primers (Additional file 2) for site-directed mutagenesis. In other words, individually, the SNPs in the promoter of YL pigs were mutated into those of LW pigs at the same site. PCR amplification was executed in a 25 μL volume, including 2 μL of dNTP mixture (2.5 mM), 0.5 μL of each forward and reverse primer (10 μM), 5 μL of 5× PrimeSTAR GXL Buffer (Mg^2+^ plus), 0.5 μL of PrimeSTAR GXL (1.25 U/μL; TaKaRa), 20 ng of pGL3-CXCL13YL plasmid, with the volume made up with H_2_O. The PCR program was as follows: 94 °C for 5 min; 18 cycles of 98 °C for 10 s, annealing temperature (Additional file 2) for 15 s, and 72 °C for 8.5 min; followed by 72 °C for 10 min. The products were treated with 1 μL *Dpn*I (Thermo Fisher Scientific), then reproduced in DH5α (Tiangen), and sequenced. The constructs obtained were named as pGL3-(−3065T), pGL3-(−2991G), pGL3-(−2473G), pGL3-(−1757C), pGL3-(−1014G), pGL3-(−712T), pGL3-(−604C), and pGL3-(−474G).

Secondly, to investigate whether or not *CXCL13* was regulated at the post-transcriptional level, RegRNA 2.0 online software [25] was used to predict that *ssc*-*miR*-*296*-*5p* probably targets the 3′-UTR of *CXCL13*. A recombinant firefly luciferase reporter vector with the *CXCL13* 3′-UTR was constructed. Gene-specific primers (3′UTR-F/R; Additional file 2) with the restriction site for *Xba*I introduced to the 5′ end were designed according to the wild-type 3′-UTR of the *CXCL13* mRNA sequence. The full-length sequence of the 3′-UTR was amplified by PCR, followed by purification. The purified products of the 3′-UTR and pGL3-Promoter vector (Promega) were digested by restriction enzyme *Xba*I (Thermo Fisher Scientific) and then purified again for the following ligation. The recombinant plasmid was sequenced and named 3′UTR-wt. Primers for site-directed mutagenesis (3′UTR-mut-F/R; Additional file 2) were designed according to the sequence of 3′UTR-wt, and the *ssc*-*miR*-*296*-*5p* target-site in *CXCL13* 3′-UTR was mutated using PrimeSTAR GXL (TaKaRa) and *Dpn*I (Thermo Fisher Scientific). The plasmid obtained was named 3′UTR-mut.

### Cell culture, transfection, and virus infection

PK15 cells were seeded at a density of 1 × 10^5^ cells per well in a 24-well plate and cultured in Dulbecco’s Modified Eagle Medium (DMEM; Gibco) with 10% FBS at 37 °C with 5% CO_2_.

To study the changes of transcriptional activity of CXCL13 promoter reporters in PK15 cells with or without PCV2 infection, 750 ng reporter construct (pGL3-CXCL13LW, pGL3-CXCL13YL, pGL3-CXCL13(−2666/+ 79), pGL3-CXCL13(−2205/+ 79), pGL3-CXCL13(−1532/+ 79), pGL3-CXCL13(−1089/+ 79), pGL3-CXCL13(−589/+ 79), pGL3-(−3065T), pGL3-(−2991G), pGL3-(−2473G), pGL3-(−1757C), pGL3-(−1014G), pGL3-(−712T), pGL3-(−604C), and pGL3-(−474G)) or pGL3-Basic, and 18.75 ng pGL4.74 vector (as an internal reference) were used to co-transfect cells using Lipofectamine^®^ LTX and Plus™ Reagent (Invitrogen), following the manufacturer’s protocol. They were assigned to three groups: non-treated, PCV2-infected, and PCV2-uninfected groups. The supernatant of non-treated groups was replaced by DMEM with 10% FBS at 6 h after transient transfection. PCV2-infected groups were inoculated with PCV2 at an MOI of 0.1 TCID_50_ and PCV2-uninfected groups were inoculated with equivalent cell lysate of PK15 at 6 h after transient transfection. The supernatant of these groups was replaced by DMEM with 2% FBS after an hour of incubation.

To evaluate the interaction between *CXCL13* and *ssc*-*miR*-*296*-*5p*, PK15 cells were co-transfected with 750 ng reporter construct (pGL3-Promoter, 3′UTR-wt, or 3′UTR-mut), and 18.75 ng pGL4.74 vector, and synthetic *ssc*-*miR*-*296*-*5p* inhibitor or inhibitor negative control at a final oligonucleotide concentration of 800 nM. The supernatant was replaced by DMEM with 10% FBS at 6 h after transient transfection.

### Luciferase reporter assay

PK15 cells transfected with different plasmids were collected at scheduled times for luciferase assays using the Dual-Luciferase Reporter Assay System, according to the manufacturer’s instruction manual (Promega). A Modulus single-tube multimode reader (Turner BioSystems, CA, USA) was used to analyze the relative luciferase expression values.

### Polymorphism analysis

The genotyping of polymorphic loci was performed in Duroc, Landrace, Yorkshire, LW, and DPL pigs. The DNA segment that covered the SNP was amplified in a 25 μL volume by PCR, containing 12.5 μL of 2 × Taq Master Mix (Novoprotein, Shanghai, China), 0.5 μL of each forward and reverse primer (10 μM; −1014SNP-F/R; Additional file 2), 1 μL of genomic DNA, and 10.5 μL of H_2_O. The PCR program was as follows: 94 °C for 1.5 min; 35 cycles of 98 °C for 20 s, 54 °C for 20 s, and 72 °C for 1 min 12 s; followed by 72 °C for 5 min. The PCR products were sent to BioSune (Jinan, China) for sequencing to determine the genotype of each individual.

### Statistical analysis

All the data in this study are presented as the mean ± standard error (SE). The differences among the groups were evaluated by one-way ANOVA and Duncan’s test using SPSS software version 17.0 (SPSS Inc., Chicago, IL, USA), and considered statistically significant at *P* < 0.05.

## Results

### Different pathological manifestations in spleen tissues of PCV2-infected YL and LW pigs

**Figure 1:**
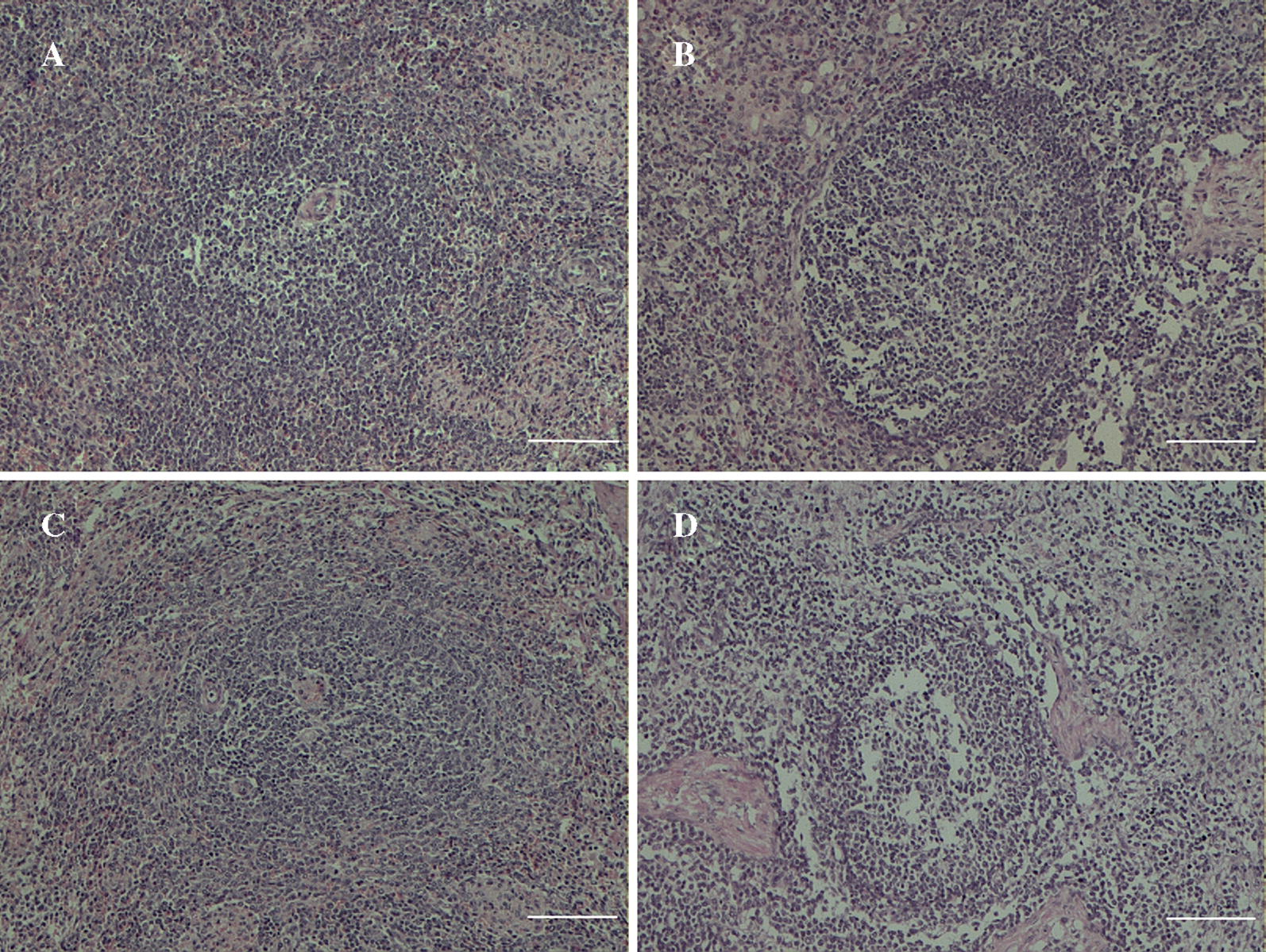
**Pathological changes in the spleen tissue of LW and YL pigs.** Tissue slices were stained by HE; the white bar indicates 100 μm. **A** Mock-infected LW pigs; **B** PCV2-infected LW pigs; **C** mock-infected YL pigs; **D** PCV2-infected YL pigs.

**Figure 2:**
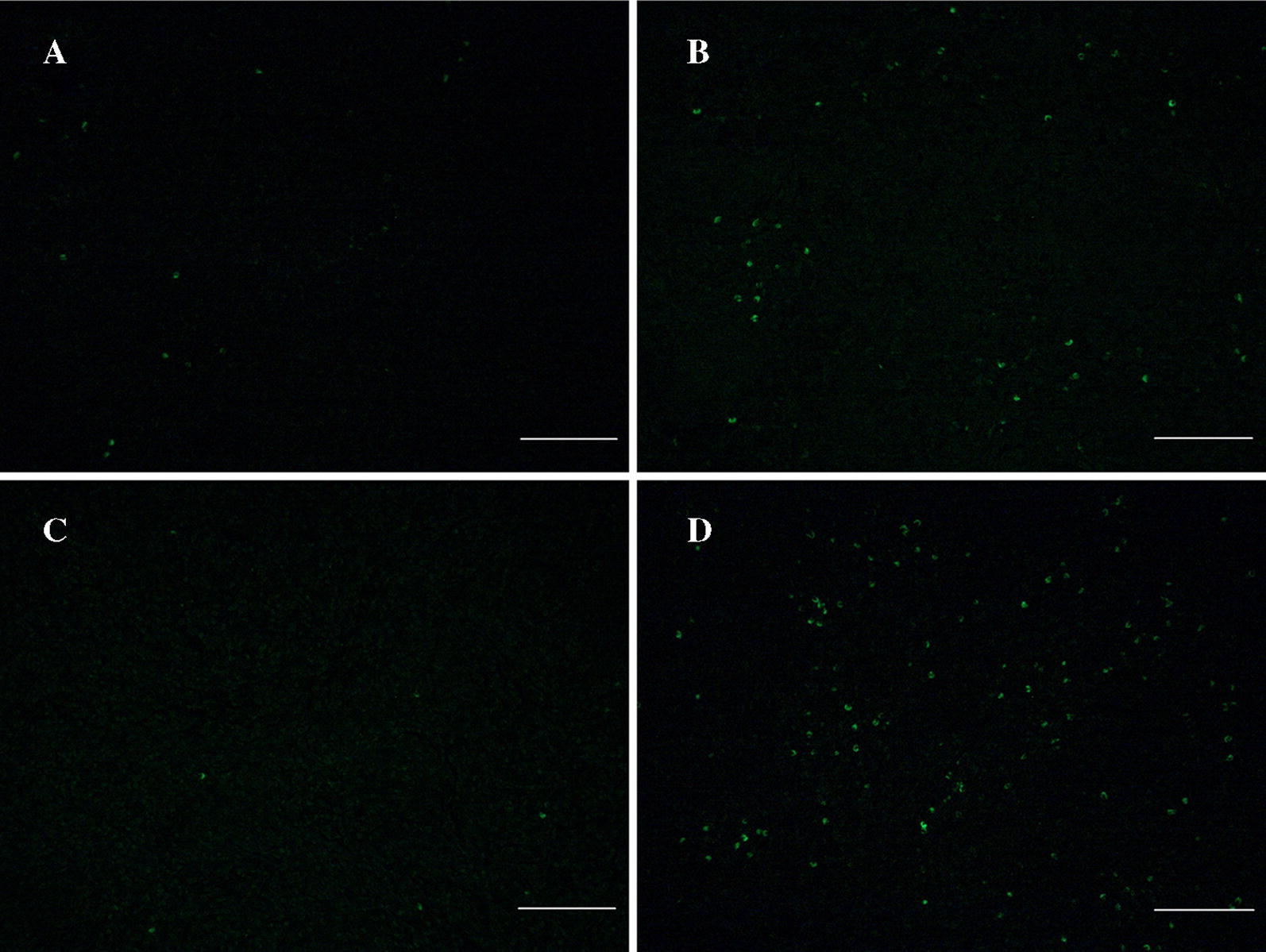
**Apoptotic cells in the spleen tissue of LW and YL pigs analyzed by TUNEL staining.** The white bar indicates 100 μm. **A** Mock-infected LW pigs; **B** PCV2-infected LW pigs; **C** mock-infected YL pigs; **D** PCV2-infected YL pigs.

In YL pigs, which are more susceptible to PCV2 infection, overt lymphocyte depletion was observed in HE-stained histopathological slides of spleens from PCV2-infected YL pigs compared with mock-infected ones (Figures 1C and D), while in LW pigs, the number of lymphocytes was only slightly decreased after PCV2 infection (Figures 1A and B). TUNEL staining also revealed an apparent increase in the number of apoptotic lymphocytes in PCV2-infected YL pigs compared to controls (Figures 2C and D), while there were no obvious changes in LW pigs (Figures 2A and B).

### Quantification of PCV2 DNA in spleen tissues

**Figure 3:**
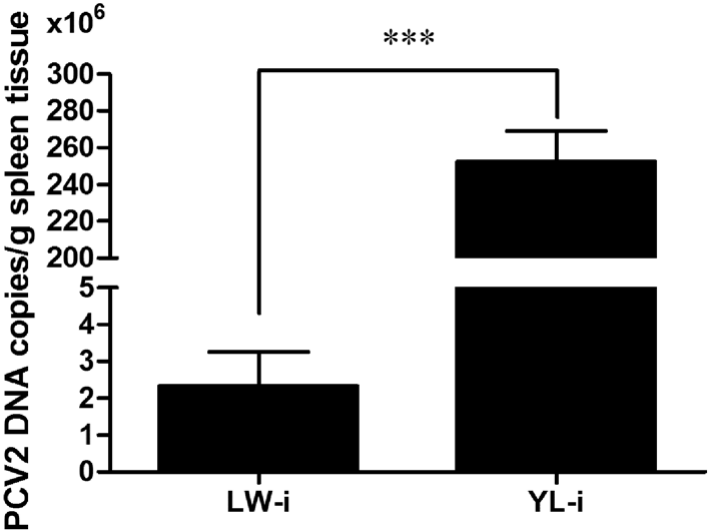
**PCV2 genomic DNA copies in spleen tissues of PCV2-infected LW and YL pigs.** The PCV2 genomic DNA copies in spleen were measured through absolute qPCR. Multiplying the value on the Y-axis by 10^6^ indicates the copy number of PCV2 genomic DNA in 1 g of spleen tissue. Data are represented as the mean value ± SE of three experiments. ***Indicates significance at the *P* value threshold level of 0.001.

The PCV2 DNA copies in porcine spleen tissues were calculated with the obtained linear formula (Ct = −3.242 × log (copy number) + 38.544) through the standard curve. As shown in Figure 3, the mean copy number of PCV2 genomic DNA in spleen tissues from PCV2-infected YL pigs was approximately 100 times higher than that from PCV2-infected LW pigs (*P* < 0.001).

### Overview of RNA-seq on the spleen tissues of YL pigs

RNA-seq on six spleen tissue samples from YL pigs generated 141 674 557 paired-end reads. The sequencing quality represented by Q30 percentage was higher than 90%. After filtering low-quality reads, 76.01–76.73% clean reads were mapped to the pig reference genome, of which 94.4–94.71% were uniquely mapped and 48.71–51.99% were perfectly matched (Additional file 3). The sequenced reads were submitted to the NCBI Gene Expression Omnibus (GEO) database under accession number [GSE112152] [26].

### Functional annotation and classification of DEGs obtained from spleen tissues of YL pigs

Ninety DEGs were identified in spleen tissues between mock- and PCV2-infected YL pigs, of which 57 were significantly up-regulated and 33 were significantly down-regulated according to the criteria of fold change > 1.5 and FDR < 0.05. The expression profiles of DEGs between mock- and PCV2-infected YL pigs were then visualized as a heat-map (Additional file 4), and the information from this analysis is detailed in Additional file 5. The classification and biological functions of the DEGs were predicted using online software DAVID and KEGG. Functional annotation analysis using DAVID showed that 11 DEGs, comprising *OAS1*, *OAS2*, *RYR2*, *GJA5*, *ADRB3*, *ADRA2C*, *CD207*, *RSAD2*, *MX2*, *CXCL13*, and *CCR1*, were significantly enriched in two molecular function gene ontology (GO) terms and five biological process GO terms (Table 1). Another 12 DEGs, comprising *PDK4*, *KLF11*, *HGF*, *PTGES3*, *MAP3K11*, *XDH*, *CYCS*, *ACTC1*, *HSPH1*, *JCHAIN*, *TRIL*, and *SOCS5*, were enriched in GO terms related to viral reproduction, cell proliferation or apoptosis, and immune system process, although these terms did not reach significant levels (Additional file 6). KEGG analysis showed that the DEGs were chiefly enriched in malaria, the adipocytokine signaling pathway, the PPAR signaling pathway, and glycine, serine, and threonine metabolism (Additional file 7).

**Table 1:** **Information about the significantly enriched GO terms**

GO ID	GO term	FDR^a^	Genes
GO:0001730	2′–5′-oligoadenylate synthetase activity	0.010373	*OAS1, OAS2*
GO:0098904	Regulation of AV node cell action potential	0.006089	*RYR2, GJA5*
GO:0098910	Regulation of atrial cardiac muscle cell action potential	0.012327	*RYR2, GJA5*
GO:0051379	Epinephrine binding	0.025933	*ADRB3, ADRA2C*
GO:0051607	Defense response to virus	0.031570	*CD207, RSAD2, OAS1, OAS2, MX2*
GO:0007267	Cell–cell signaling	0.039087	*ADRB3, CXCL13, CCR1, ADRA2C*
GO:0086005	Ventricular cardiac muscle cell action potential	0.036724	*RYR2, GJA5*

^a^FDR: false discovery rate.

### Validation of twelve DEGs related to cell proliferation or apoptosis in LW and YL pigs

**Figure 4:**
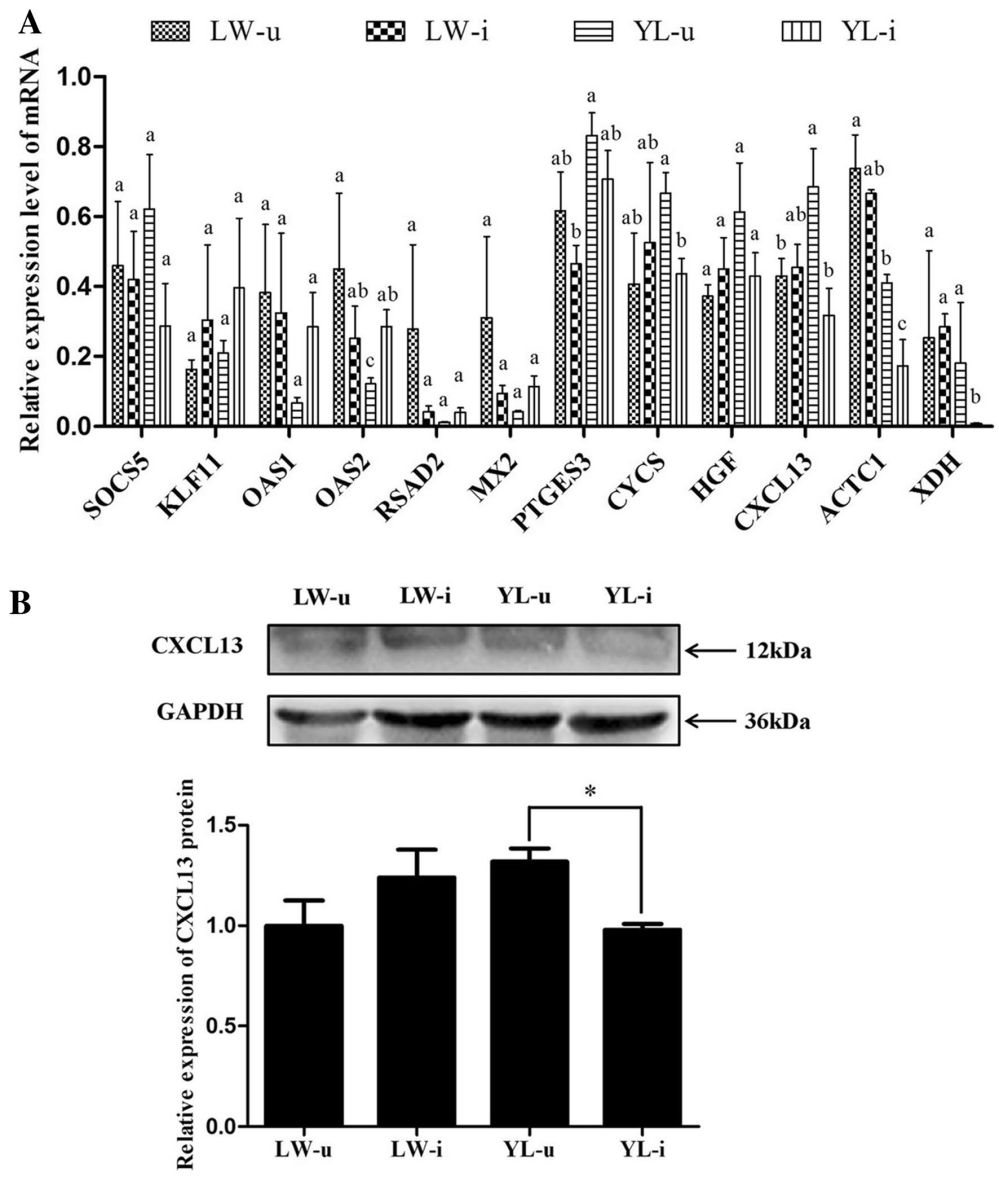
**Twelve DEGs validated by RT-qPCR and expression changes of CXCL13 protein in porcine spleens after PCV2 infection. A** Twelve candidate genes validated by RT-qPCR in LW and YL pigs. The *HPRT1* gene was used as an internal control. Data are represented as the mean value ± SE of three experiments. Different lowercase letters indicate significance at the *P* value threshold level of 0.05. **B** Expression changes of CXCL13 protein in porcine spleens after PCV2 infection. The *GAPDH* gene was used as an internal control. LW-u, mock-infected LW pigs; LW-i, PCV2-infected LW pigs; YL-u, mock-infected YL pigs; YL-i, PCV2-infected YL pigs. Data are represented as the mean value ± SE of three experiments. *Indicates significance at the *P* value threshold level of 0.05.

The expression pattern of 12 DEGs that are related to cell proliferation or apoptosis was validated by RT-qPCR (Figure 4A and Table 2). Both RNA-seq and RT-qPCR revealed that *OAS2*, *CYCS*, *CXCL13*, *ACTC1* and *XDH* were significantly up- or down-regulated in response to PCV2 infection in YL pigs (*P* < 0.05). The other genes, except *SOCS5*, all displayed a consistent, but not significant, change in YL pigs. However, the expression level of all the above DEGs showed no significant difference between PCV2-infected and mock-infected LW pigs. Furthermore, the protein expression of CXCL13 was detected by Western blotting, and the result showed a decrease in CXCL13 protein in the spleen tissues of YL pigs (*P* < 0.05) but not in LW pigs after PCV2 challenge (Figure 4B).

**Table 2:** **Detailed information of the twelve DEGs selected from the results of RNA-seq**

Gene	Description	FDR	Fold change^a^
SOCS5	Suppressor of cytokine signaling 5	0.00548433	3.72083089
KLF11	Kruppel like factor 11	0.00052139	3.39527881
OAS1	2′–5′-oligoadenylate synthetase 1	0.03365008	2.51892472
OAS2	2′–5′-oligoadenylate synthetase 2	0.03414863	2.34579177
RSAD2	Radical S-adenosyl methionine domain containing 2	0.00179287	1.92045179
MX2	Myxovirus resistance protein 2	0.02527284	1.81462201
PTGES3	Prostaglandin E synthase 3	0.02953347	−1.65501931
CYCS	Cytochrome c, somatic	0.04789901	−1.65870931
HGF	Hepatocyte growth factor	0.02886981	−1.76664727
CXCL13	C–X–C motif chemokine ligand 13	0.00019681	−1.95065761
ACTC1	Actin, alpha, cardiac muscle 1	0.00103903	−2.27412576
XDH	Xanthine dehydrogenase	0.00013513	−6.35848339

^a^Fold change: PCV2-infected group/mock-infected group.

### CXCL13 can suppress lymphocyte apoptosis during PCV2 infection

**Figure 5:**
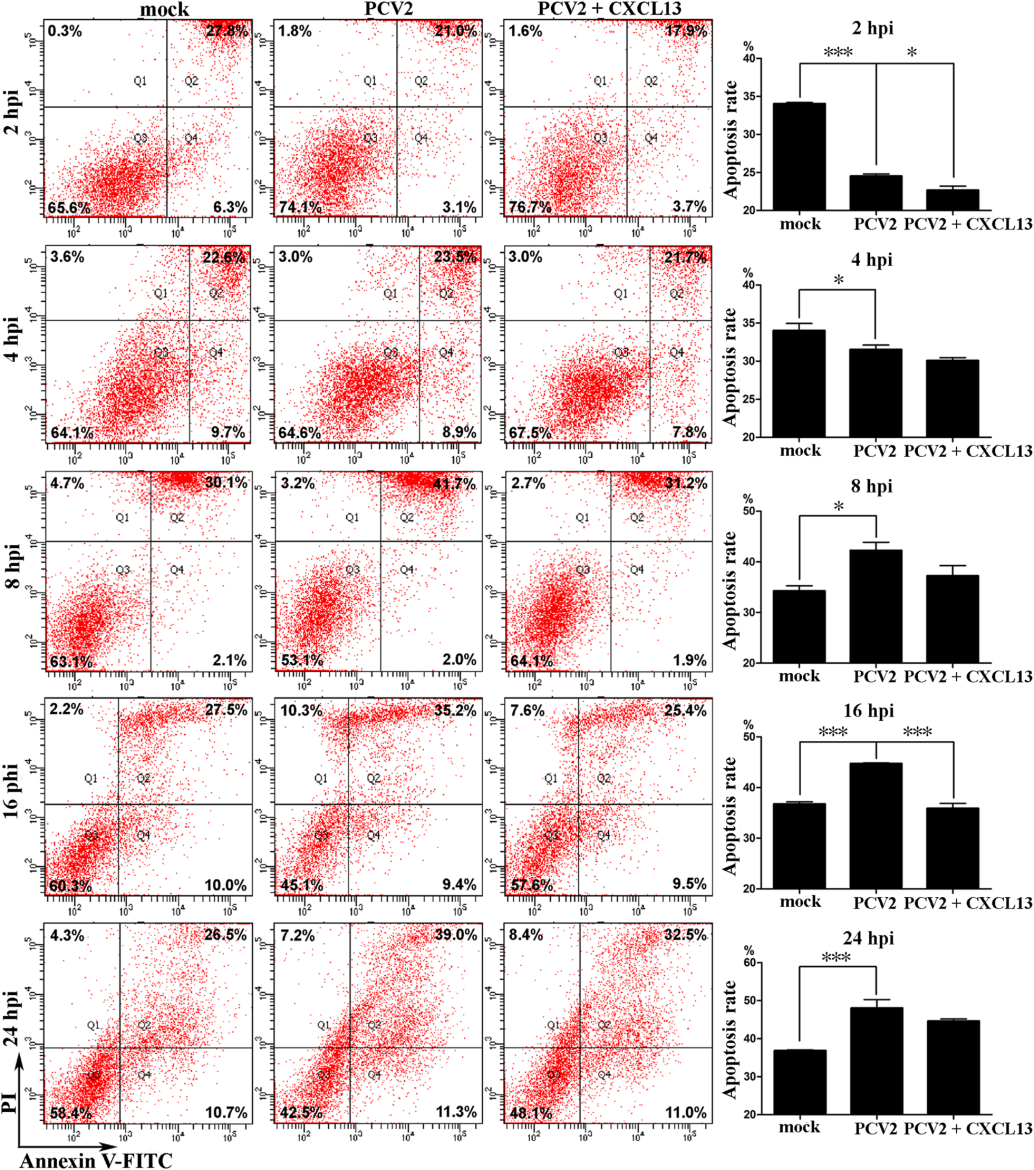
**CXCL13 can suppress the apoptosis of murine splenocytes during PCV2 infection.** Splenocytes were freshly isolated from murine spleen and treated with PCV2 at an MOI of 0.1 TCID_50_ alone or along with CXCL13 (1 μg/mL). The cells were analyzed by flow cytometry for PI (*y* axis) and FITC-conjugated annexin V (*x* axis) at 2, 4, 8, 16, and 24 hpi after treatment. The total percentages of PI^−^ annexin V^+^ cells (Q4) and PI^+^ annexin V^+^ cells (Q2) indicate the apoptosis rate. The nine scatter plots shown on the left are from a single experiment, which was representative of three separately performed experiments. The bar graphs shown on the right indicate mean value ± SE of three experiments. * and *** indicate significance at the *P* value threshold levels of 0.05 and 0.001, respectively.

To determine whether the CXCL13 protein affects PCV2-induced apoptosis of spleen lymphocytes, the apoptotic rate of PCV2-infected murine splenocytes in the presence or absence of murine CXCL13 protein was measured using the annexin V and propidium iodide (PI) double-staining method by flow cytometry at 2, 4, 8, 16, and 24 hpi, and splenocytes treated with equivalent PK15 cell lysate served as a negative control. As demonstrated in Figure 5, the apoptotic rate induced by PCV2 alone was significantly lower than that of the negative control at 2 and 4 hpi (*P* < 0.001 and *P* < 0.05), whereas it was significantly higher than the negative control at 8, 16, and 24 hpi (*P* < 0.05, *P* < 0.001, and *P* < 0.001), suggesting that PCV2 may activate endogenous antiapoptotic processes during the early stage of infection, but stimulate splenocytes apoptosis during the late stage of infection. However, when the PCV2-infected splenocytes were co-treated with CXCL13 protein, the apoptotic rate significantly declined compared with that of splenocytes infected with PCV2 alone at 2 and 16 hpi (*P* < 0.05 and *P* < 0.001). At other time points, the apoptotic rate of PCV2 and CXCL13 co-treated splenocytes was also lower than that of splenocytes infected with PCV2 alone, but not significantly (*P* > 0.05). These results indicate that the CXCL13 protein plays an important role in suppressing lymphocyte apoptosis during PCV2 infection. We suppose that the decline of CXCL13 expression in YL pigs after PCV2 infection is the cause of lymphocyte depletion and subsequently analyzed the regulatory mechanism of CXCL13 in pigs.

### PCV2 infection can enhance the transcriptional activity of the *CXCL13* promoter cloned from LW pigs but not YL pigs

**Figure 6:**
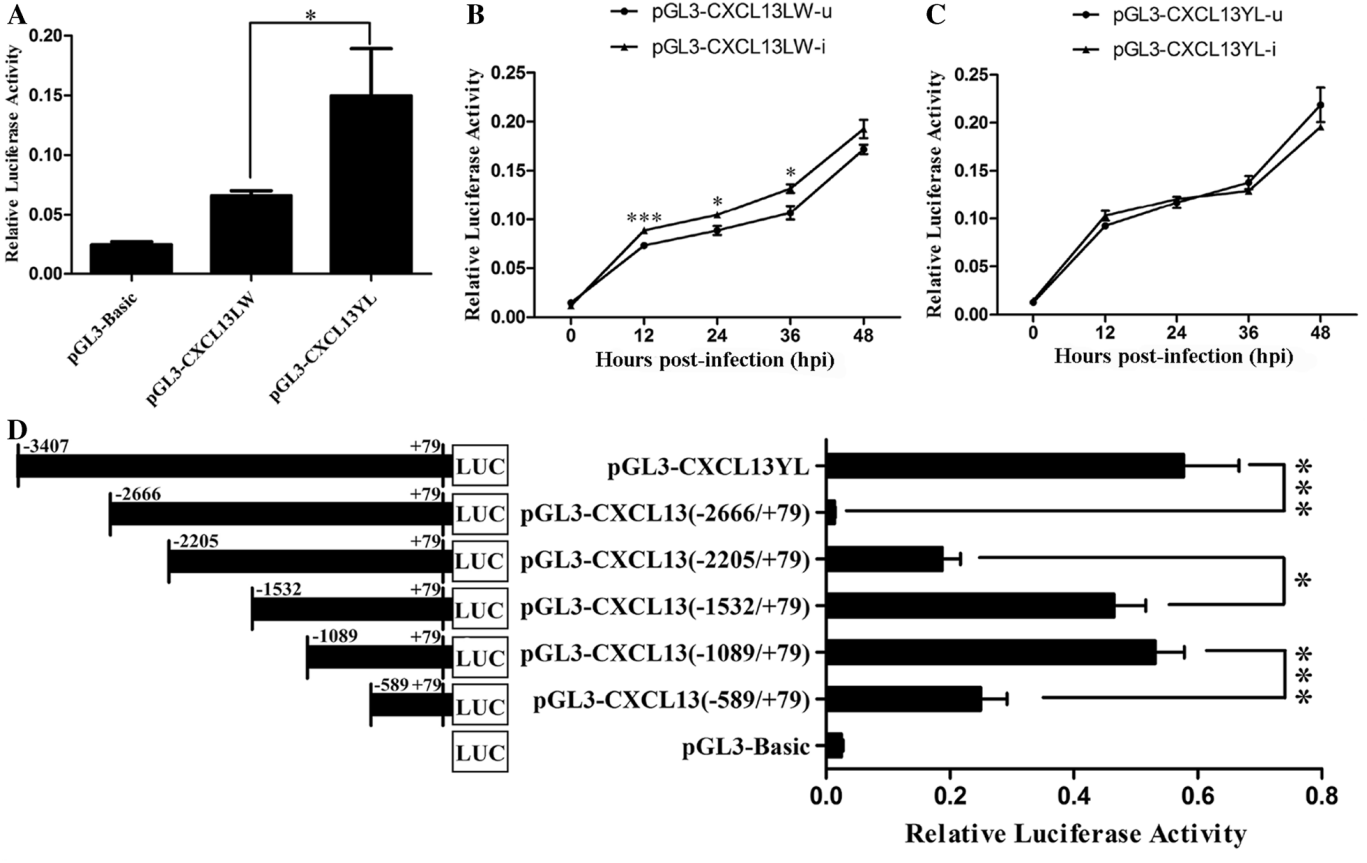
**Transcriptional activity of the** ***CXCL13*** **promoter cloned from LW and YL pigs. A** Luciferase activities of pGL3-CXCL13LW and pGL3-CXCL13YL in PK15 cells at 24 h post-transfection. Luciferase activities of pGL3-CXCL13LW (**B**) or pGL3-CXCL13YL (**C**) in PK15 cells with and without PCV2 infection. PCV2-infected groups (pGL3-CXCL13LW-i and pGL3-CXCL13YL-i) were inoculated with PCV2 at an MOI of 0.1 TCID_50_ and PCV2-uninfected groups (pGL3-CXCL13LW-u and pGL3-CXCL13YL-u) were treated with equivalent PK15 cell lysate at 6 h post-transfection. Luciferase activity was detected at 0, 12, 24, 36, and 48 hpi. **D** Detailed delineation of the promoter regions of the porcine *CXCL13* gene by 5′ deletion analysis. The left panel shows a schematic diagram for firefly luciferase reporter constructs containing the indicated genomic sequences upstream of *CXCL13*. The right panel shows the luciferase activity of the corresponding deletion construct. At least three separate experiments were performed with each plasmid DNA preparation. Data are represented as the mean value ± SE of three experiments. * and *** indicate significance at the *P* value threshold levels of 0.05 and 0.001, respectively.

To verify whether the polymorphism in the porcine *CXCL13* promoter affects its transcription level, a 3486 bp fragment spanning the 5′-flanking region of *CXCL13* (from −3407 to +79) was cloned from LW and YL pigs and used in a luciferase assay. As shown in Figure 6A, in the absence of PCV2, the promoter fragment of YL pigs (pGL3-CXCL13YL) showed significantly higher transcriptional activity than that of LW pigs (pGL3-CXCL13LW, *P* < 0.05). This in vitro result corresponds well to the in vivo result mentioned above (Figure 4A), in which the mRNA expression of *CXCL13* in the spleen tissue of mock-infected YL pigs is significantly higher than that of mock-infected LW pigs. After PCV2 infection, the activity of pGL3-CXCL13LW increased significantly from 12 to 36 hpi (*P* < 0.001 and *P* < 0.05) compared with that in mock-infected controls (Figure 6B); however, the activity of pGL3-CXCL13YL was similar to that of the mock-infected controls, and even lower at 36 and 48 hpi (Figure 6C). These results indicated that, compared with YL pigs, the nucleotide differences in the *CXCL13* promoter of LW pigs may enhance its transcriptional activity in response to PCV2 infection by altering the interaction between the *cis*-acting elements and the corresponding transcription factors.

### Polymorphisms in the promoter region of porcine *CXCL13*

**Figure 7:**
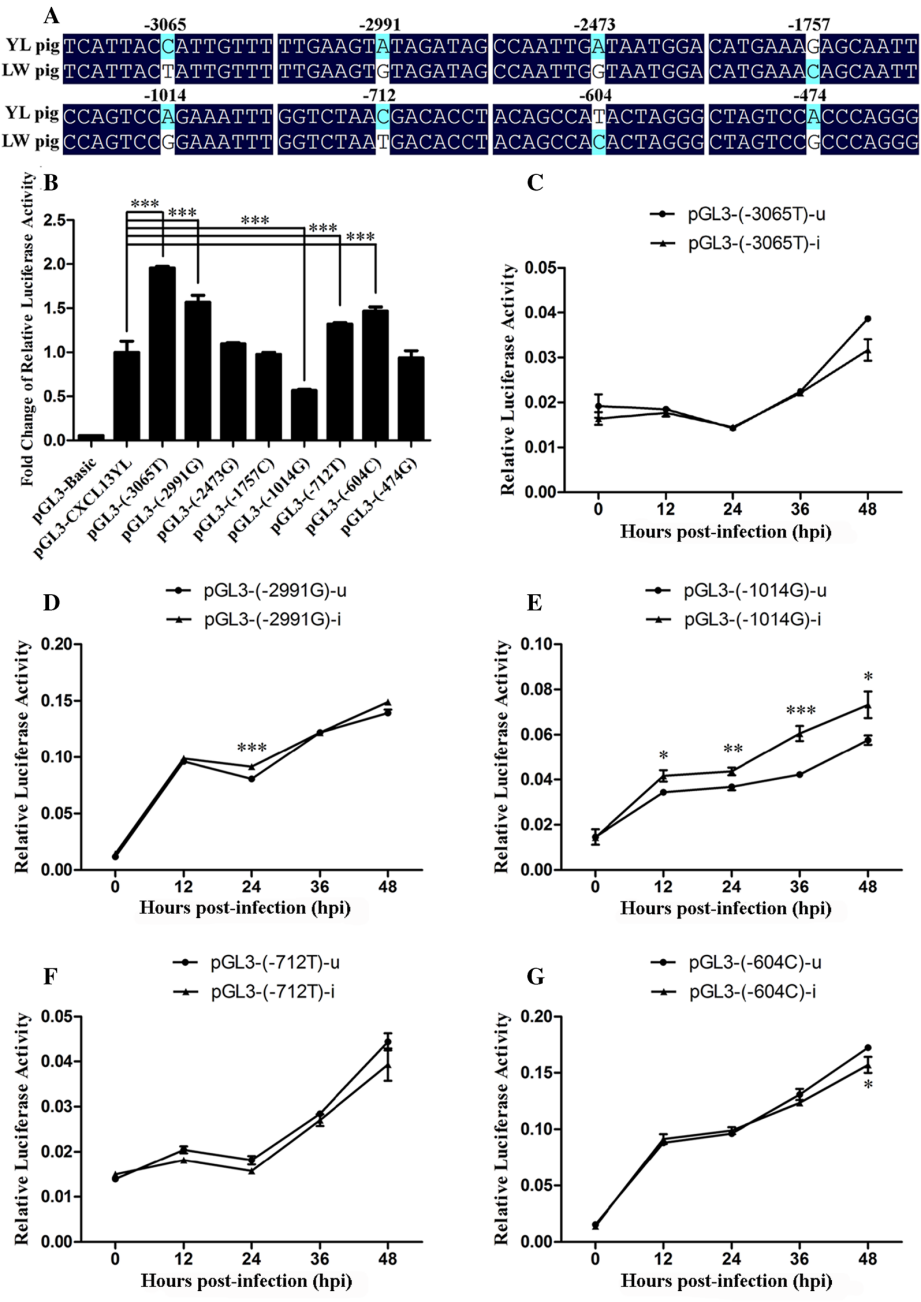
**Effect of each SNP on the transcriptional activity of the** ***CXCL13*** **promoter. A** Eight SNPs discovered in the critical promoter region of porcine *CXCL13*. **B** Luciferase activities of pGL3-CXCL13YL and site-directed mutants of pGL3-CXCL13YL in PK15 cells at 48 h post-transfection. **C**–**G** Luciferase activities of each site-directed mutant of pGL3-CXCL13YL in PK15 cells with and without PCV2 infection. PCV2-infected groups (pGL3-(−3065T)-i, pGL3-(−2991G)-i, pGL3-(−1014G)-i, pGL3-(−712T)-i, and pGL3-(−604C)-i) were inoculated with PCV2 at an MOI of 0.1 TCID_50_, and PCV2-uninfected groups (pGL3-(−3065T)-u, pGL3-(−2991G)-u, pGL3-(−1014G)-u, pGL3-(−712T)-u, and pGL3-(−604C)-u) were treated with equivalent PK15 cell lysate at 6 h post-transfection. Luciferase activity was detected at 0, 12, 24, 36, and 48 hpi. Each bar represents the mean value ± SE of three experiments. *, **, and *** indicate significance at the *P* value threshold levels of 0.05, 0.01, and 0.001, respectively.

Critical promoter regions that regulate the transcriptional activity of the porcine *CXCL13* gene were identified by a luciferase assay of serial 5′ deletion constructs. Deletions from −3407 bp to −2666 bp, −1089 bp to −589 bp, and −2205 bp to −1532 bp caused a pronounced reduction or increase in the luciferase activity, suggesting the existence of potential positive or negative transcription regulatory elements in these fragments (Figure 6D). Eight SNPs were found in these regions by alignment of sequences from LW and YL pigs: −3065 T (LW) > C (YL), −2991 G (LW) > A (YL), −2473 G (LW) > A (YL), −1757 C (LW) > G (YL), −1014 G (LW) > A (YL), −712 T (LW) > C (YL), −604 C (LW) > T (YL), and −474 G (LW) > A (YL) (Figure 7A).

### Effect of the −1014 G (LW) > A (YL) site on transcriptional activity of the *CXCL13* promoter

To evaluate the effect of the SNPs on the promoter activity of *CXCL13*, the nucleotide at each SNP site in pGL3-CXCL13YL was mutated into the corresponding nucleotide in pGL3-CXCL13LW. The luciferase activity of pGL3-(−3065T), pGL3-(−2991G), pGL3-(−712T), and pGL3-(−604C) was significantly higher than that of pGL3-CXCL13YL (*P* < 0.001), whereas the luciferase activity of pGL3-(−2473G), pGL3-(−1757C), and pGL3-(−474G) showed no significant difference compared with that of pGL3-CXCL13YL. Notably, the construct pGL3-(−1014G) displayed significantly lower luciferase activity than pGL3-CXCL13YL, suggesting that the −1014 G (LW) > A (YL) site may be related to the differential expression of *CXCL13* between mock-infected LW and YL pigs (Figure 7B).

We then validated the role of each SNP on the transcriptional activity of the *CXCL13* promoter in response to PCV2 infection. Compared with the mock-infected control, there was no significant change in the luciferase activity of pGL3-(−3065T), pGL3-(−712T), pGL3-(−604C), and pGL3-(−2991G) at each time point, except the 24 hpi sample of pGL3-(−2991G), at which time the luciferase activity increased remarkably compared with the mock-infected control (*P* < 0.001), as well as the 48 hpi of pGL3-(−604C), at which time point the luciferase activity declined significantly compared with the mock-infected control (*P* < 0.05) (Figures 7C, D, F, and G). Interestingly, consistent with the above observation that the luciferase activity of pGL3-CXCL13LW increased significantly in response to PCV2 infection (Figure 6B), the luciferase activity of pGL3-(−1014G) in PCV2-infected groups was always dramatically higher than that of mock-infected controls from 12 hpi to 48 hpi (*P* < 0.05; Figure 7E), indicating that the G (LW) > A (YL) at site −1014 is crucial for the differential expression of *CXCL13* between LW and YL pigs after PCV2 challenge.

### Polymorphism of the −1014 G (LW) > A (YL) site among different pig breeds

The polymorphism of G > A at site −1014 was detected in five purebred pig populations, including three western breeds, Duroc, Landrace, and Yorkshire, and two Chinese indigenous breeds, LW and Dapulian (DPL). As shown in Table 3, in LW and DPL populations, the allele G was predominant, and the homozygote GG existed in more than 95% of the samples; whereas the allele A was rare (about 2%) and appeared only in the heterozygous form (GA). Compared with Chinese breeds, the frequency of allele A was higher in all three western breeds, in which the homozygote AA was found to be present in more than 5% of the samples and the heterozygote GA became the relatively dominant genotype. Allelic and genotype frequencies of all five populations conformed to the Hardy–Weinberg equilibrium. Based on the above observation that the allele G played a positive role in stimulating the expression of the *CXCL13* after PCV2 infection, we found that the distributions of alleles G and A in different pig breeds were consistent with the breeds’ differences in resistance to PCV2.

**Table 3:** **Polymorphism of SNP −1014 G > A in different pig breeds**

Breed	No.	Genotype frequency			Allelic frequency		χ^2^	*P* value
		GG	GA	AA	G	A		
LW pig	46	95.65% (*n* = 44)	4.35% (*n* = 2)	0.00% (*n* = 0)	97.83%	2.17%	0.0227	0.8802
DPL pig	49	95.92% (*n* = 47)	4.08% (*n* = 2)	0.00% (*n* = 0)	97.96%	2.04%	0.0213	0.8841
Duroc	46	34.78% (*n* = 16)	58.70% (*n* = 27)	6.52% (*n* = 3)	64.13%	35.87%	3.4992	0.0614
Landrace	51	17.65 (*n* = 9)	56.86% (*n* = 29)	25.49% (*n* = 13)	46.08%	53.92%	1.0619	0.3028
Yorkshire	56	41.07% (*n* = 23)	53.57% (*n* = 30)	5.36% (*n* = 3)	67.86%	32.14%	2.9129	0.0879

### *CXCL13* is directly targeted by *ssc*-*miR*-*296*-*5p*

**Figure 8:**
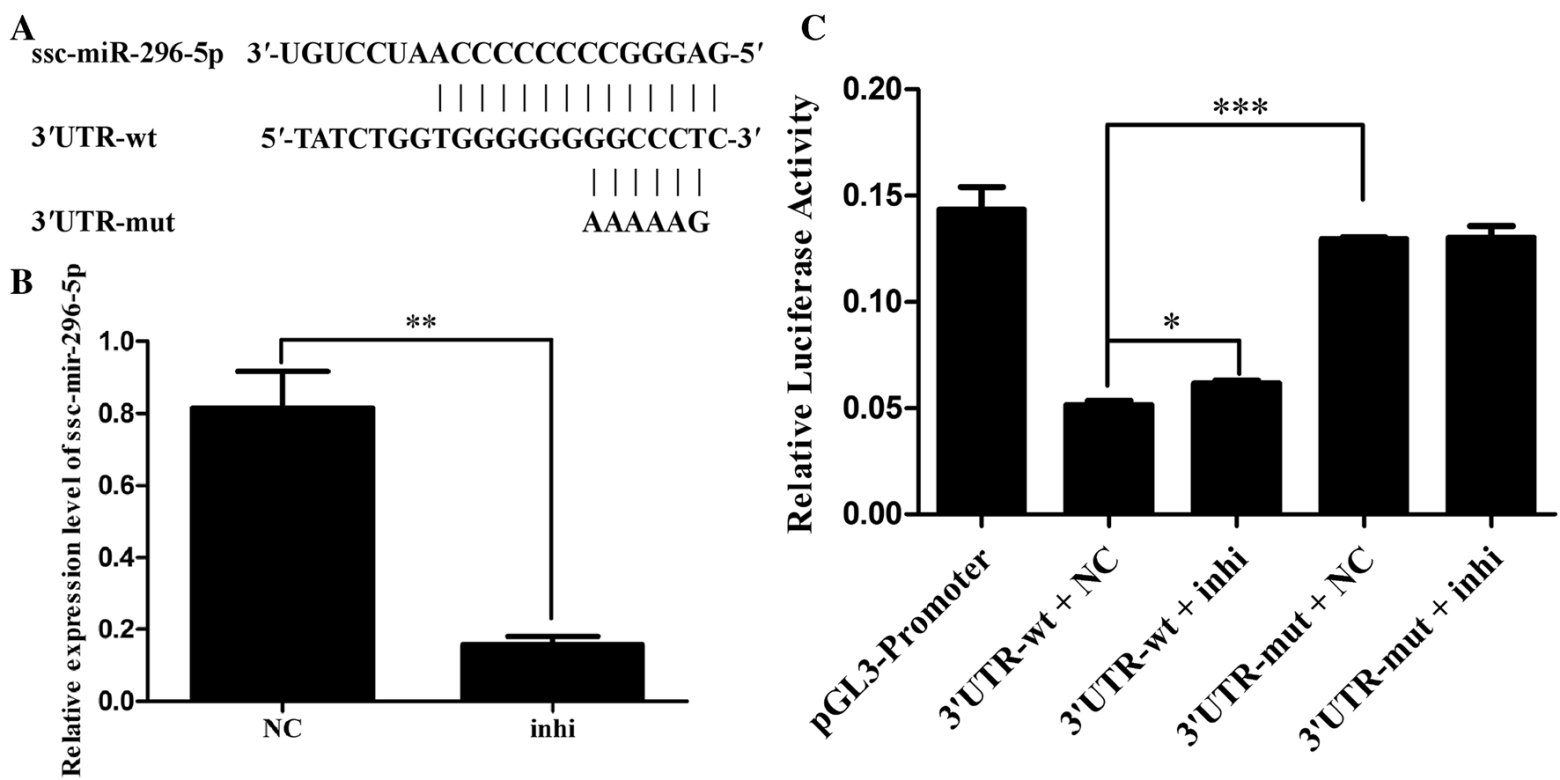
***CXCL13*** **is a direct target of** ***ssc*-*miR*-*296*-*5p***. **A** Diagram of the predicted targeting site within the 3′-UTR of *CXCL13* (3′UTR-wt). The mutated 3′-UTR (3′UTR-mut) contains mutated sequence that is not complementary to the seed sequence (CCGGGAG) of *ssc*-*miR*-*296*-*5p*. **B** Expression of *ssc*-*miR*-*296*-*5p* in PK15 cells at 24 h post-transfection with 800 nM inhibitor (inhi) or negative control (NC). **C** Luciferase activity of the constructs harboring the wild-type or mutant 3′-UTR of the *CXCL13* gene in PK15 cells with 800 nM *ssc*-*miR*-*296*-*5p* inhibitor or negative control. Data are represented as the mean value ± SE of three experiments. *, **, and *** indicate significance at the *P* value threshold levels of 0.05, 0.01, and 0.001, respectively.

We further investigated whether there is any difference in the post-transcriptional regulation of *CXCL13* between LW and YL pigs. According to the bioinformatic prediction of RegRNA 2.0, *ssc*-*miR*-*296*-*5p* was the only miRNA that targeted to the 3′-UTR of the porcine *CXCL13* gene (Figure 8A). To verify the target relationship between *ssc*-*miR*-*296*-*5p* and *CXCL13*, the expression level of *ssc*-*miR*-*296*-*5p* in spleens was measured by RT-qPCR. Compared with mock-infected controls, the expression of *ssc*-*miR*-*296*-*5p* increased by 27% in the spleen tissues of PCV2-infected YL pigs, but merely increased by 8% in PCV2-infected LW pigs (Table 4), demonstrating that the expression pattern of *ssc*-*miR*-*296*-*5p* was opposite to that of *CXCL13* in mock- and PCV2-infected YL pigs. Subsequently, the luciferase assay was utilized to determine the target relationship between *ssc*-*miR*-*296*-*5p* and *CXCL13* in PK15 cells. As there is a high constitutive expression of *ssc*-*miR*-*296*-*5p* in PK15 cells, we first showed that the expression of *ssc*-*miR*-*296*-*5p* dramatically declined when the PK15 cells were transfected with *ssc*-*miR*-*296*-*5p* inhibitor (*P* < 0.01; Figure 8B). Then, either the 3′UTR-wt construct, which harbors the wild-type 3′-UTR of *CXCL13,* or the 3′UTR-mut construct, which contains the mutant 3′-UTR of *CXCL13* (Figure 8A), was used to transfect PK15 cells and the luciferase activity was analyzed. As expected, the luciferase activity of the 3′UTR-mut was significantly higher than that of the 3′UTR-wt (*P* < 0.001; Figure 8C). Meanwhile, co-transfection of the 3′UTR-wt or 3′UTR-mut with the *ssc*-*miR*-*296*-*5p* inhibitor showed that the *ssc*-*miR*-*296*-*5p* inhibitor significantly elevated the luciferase activity of the 3′UTR-wt (*P* < 0.05), but not of the 3′UTR-mut. These results suggest that the expression of *CXCL13* is also regulated by *ssc*-*miR*-*296*-*5p* at the post-transcriptional level, and *ssc*-*miR*-*296*-*5p* is likely to play a regulatory role in the differential expression of *CXCL13* between LW and YL pigs after PCV2 infection.

**Table 4:** **Expression change of** ***ssc*** **-** ***miR*** **-** ***296*** **-** ***5p*** **in porcine spleens after PCV2 infection**

Gene	LW-u	LW-i	Fold change (LW-i vs LW-u)	YL-u	YL-i	Fold change (YL-i vs YL-u)
Ssc-miR-296-5p	0.425 ± 0.083	0.461 ± 0.016	1.08	0.554 ± 0.092	0.705 ± 0.109	1.27

The expression pattern of *ssc*-*miR*-*296*-*5p* was confirmed by RT-qPCR in LW and YL pigs. The 5S rRNA was used as an internal control. Data are represented as the mean value ± SE of three experiments.

## Discussion

To date, evidence of breed-dependent differences in susceptibility to PCV2 has mainly come from the comparison of phenotypic differences after PCV2 infection, including the clinical evaluation, histologic examination, and quantification of PCV2 [12–15]. However, few studies have been focused on the differential gene expression after PCV2 infection between pig breeds, especially between the lean-meat pig breeds and Chinese indigenous pig breeds. Our previous studies compared the experimental infection responses of LW and YL pigs to PCV2-SD, and found that LW pigs had less serious clinical symptom and lung lesions, as well as a lower viral load, than YL pigs, suggesting that the LW pigs had a stronger resistance or tolerance to PCV2 infection than YL pigs. Further comparison of the lung transcriptome of infected LW and YL pigs indicated that the higher expression of *SERPINA1* may effectively inhibit the inflammation reaction and lessen pathological damage in the lung tissue of the PCV2-infected LW pigs [11].

In addition to lung lesions, lymphoid depletion and histiocytic replacement in lymphoid tissues, which leads to immunosuppression in pigs, are also characteristic lesions of PCV2 infection and PCVAD [3, 27]. In the present study, we found, by both HE- and TUNEL staining of infected spleen tissues, obvious depletion of lymphocytes in the spleen sections of PCV2-infected YL pigs, but not in those of LW pigs. In accordance with this, the copy number of PCV2 DNA in the spleen tissues was significantly higher in PCV2-infected YL pigs than in PCV2-infected LW pigs. A previous study revealed that the expression of immune-related genes in inguinal lymph nodes, such as chemokine receptors (*CCR2*, *CCR3*, *CCR5*, *CXCR3* and *CXCR4*), T and/or B cell development and/or activation related genes (*TNFSF13B*, *IL*-*4R*, *CD27*, *CD45*, *CD71*, *CD81*, *CD82* and *CD86*) and cell apoptosis related genes (*Bcl*-*x*, *Fas* and *Bcl*-*6*), were up-regulated at low PCV2 load, but the up-regulated level tended to decrease or turned into down-regulation as the PCV2 load increased [28]. Once virus load reached the full capacity in host cells, PCV2 may manipulate the expression of apoptosis-related genes to facilitate cell death [29]. These reports were consistent with that a higher virus load was accompanied with higher lymphocyte depletion in the spleen tissue of PCV2-infected YL pigs, as is shown in this study.

To uncover host genes underlying lymphocyte depletion in spleen tissues that is caused by PCV2 infection, we compared differences in transcriptome of spleen tissues between mock-infected and PCV2-infected YL pigs and identified 90 DEGs. Transcriptome analysis on the spleen tissues of mock-infected and PCV2-infected LW pigs, which showed no obvious lesions, could definitely provide more information for the purpose of correlating gene expression and alterations in spleen, and could serve an additional control to study the effect of PCV2 in the spleen of YL pigs. We overcame this inadequacy by testing 12 of these DEGs by RT-qPCR in both mock-infected and PCV2-infected YL and LW pigs. We will further analyze transcriptomic differences on non-infected and PCV2-infected LW pigs and uncover differences between mock-infected and PCV2-infected YL and LW pigs.

In this study, six spleen samples from three mock-infected and three PCV2-infected YL pigs (with obvious lymphocyte depletion), respectively, were used for RNA-seq. However, correlation analysis between samples revealed that the R^2^ values between one sample and the other two in both non-infected and PCV2-infected groups was lower, which is due to individual variations. In bioinformatics analysis, only two samples with higher R^2^ values in each group were selected to obtain more DEGs. Subsequently, the DEGs were validated with RT-qPCR in spleen tissues from four pig groups, four individuals for each group: non-infected YL and LW groups and PCV2-infected YL and LW groups. For YL pigs, eight spleen samples including the six samples for RNA-seq were used; and for LW pigs, four spleen samples were sampled from mock-infected and PCV2-infected LW pigs, respectively. Among the 12 genes selected for validation, five genes including CXCL13 were significantly changed in YL pigs, while the expression of the other seven genes was not significantly changed, which is likely caused by individual variations.

The mechanism by which PCV2 causes lymphoid depletion has yet to be identified definitively. The controversy mainly lies in whether the lymphocyte depletion results from apoptosis or reduced proliferation. For this reason, we focused on the DEGs that take part in cell apoptosis or proliferation, as well as in the immune process. We finally chose *CXCL13* as a candidate gene for further investigation, because it matched our filter criteria for DEGs, and most importantly, it was differentially expressed between PCV2-infected LW and YL pigs, both at the levels of mRNA and protein.

CXCL13, also known as B cell-attracting chemokine-1 (BCA-1) or B-lymphocyte-chemoattractant (BLC), is a member of the CXC chemokine family, which includes proteins that play important roles in the activation and recruitment of leukocytes [30, 31]. At present, most studies about the function of CXCL13 have been based on humans and mice, but few have been based on livestock. Under normal physiological conditions, CXCL13 is mainly produced by follicular dendritic cells (FDCs) in the germinal center (GC) of the secondary lymphoid organs [32–34]. CXCL13, coupled with its receptor CXCR5, is essential for the recruitment of B cells into the light zone of GC, where the B cells are selected to differentiate into memory B cells or to undergo apoptosis through interaction with GC T cells and FDCs [35, 36]. *CXCL13*^−*/*−^ mice have a severe but partial absence of peripheral lymphoid organs, as well as failure to form B-cell follicles [36]. By contrast, in inflammatory disorders, ectopic expression of CXCL13 leads to the local development of GC-like tissue in inflamed tissues [37–39]. While CXCL13 is crucial for B cell trafficking and GC formation, it has also been linked to the regulation of cell apoptosis. CXCL13 and CCL19 together can inhibit the TNFα-mediated apoptosis of the CD23^+^ CD5^+^ B cell lineage, which is the commonest malignant cell type in acute and chronic lymphocytic leukemia [40]. A recent in vitro study also illustrated that CXCL13 can significantly attenuate the degree of lipid-induced apoptosis in macrophages, primary monocytes, and vascular smooth muscle cells [41]. In the present study, using the mouse model, which has been widely accepted as an infection model for elucidating the interaction between PCV2 and host [42], the anti-apoptotic role of the CXCL13 protein was evaluated in PCV2-infected splenocytes in vitro. As expected, the proportion of apoptotic splenocytes declined at every time point after the exogenous CXCL13 was added into the medium, and this anti-apoptotic effect was more pronounced at 4 and 16 hpi. This finding extended our understanding that CXCL13 could down-regulate the virus-induced apoptosis in spleen lymphocytes. Therefore, the stable expression of CXCL13 before and after PCV2 infection might be responsible for the minor lymphocyte apoptosis in PCV2-infected LW pigs. By contrast, the significant decline of CXCL13 after PCV2 infection is likely to have contributed to the severe lymphocyte apoptosis in PCV2-infected YL pigs.

The investigation on the molecular mechanism of the differential expression of *CXCL13* between LW and YL pigs showed that the SNP −1014 G > A in the 5′ regulatory region of *CXCL13* may serve as a functional DNA marker associated with PCV2 resistance. Then, we used MatInspector software to search for potential transcription binding sites in sequences harboring this SNP. The search revealed that the G nucleotide at the −1014 site created an extra binding site for two transcription factors, the nuclear factor of activated T-cells 5 (NFAT5) and the E-twenty-six (ETS) transcription factor (ELK1). NFAT5, also known as TonEBP or OREBP, is a member of the Rel family of transcription factors. Accumulating evidence indicates that, in addition to a pivotal role in protecting mammalian cells against hyperosmotic stress, NFAT5 also has a variety of other functions, especially in the stimulation of expression of multiple cytokines and immune receptors in leukocytes during physiological and pathophysiological processes [43–45]. ELK1 belongs to the ternary complex factor (TCF) subfamily of ETS-domain transcription factors. Recent findings show that ELK1 mainly acts in a dynamic fashion with other ETS transcription factors to control the expression of many immediate-early (IE) genes encoding transcription factors, signaling pathway regulators, and RNA-interacting proteins involved in cell proliferation, the cell cycle, and apoptosis [46, 47]. Whether or not the NFAT5 and/or ELK1 can bind to the G allele and induce the expression of *CXCL13* needs to be further investigated.

We noticed that the decrease of CXCL13 in PCV2-infected YL pigs was not only at the mRNA level but also at the protein level. So, in the last part of this study, we investigated whether the differential expression of CXCL13 between LW and YL pigs is also regulated by differential expression of miRNAs, which act as the key regulators of gene expression at the post-transcriptional level. By analyzing the 3′-UTR sequence of porcine *CXCL13*, we found a well-matched target site for *ssc*-*miR*-*296*-*5p*, and this site is conserved in both LW and YL pigs. Subsequent luciferase reporter assays further proved that *ssc*-*miR*-*296*-*5p* had the ability to mediate repression of luciferase via the *CXCL13* 3′-UTR. Due to the greater increase of *ssc*-*miR*-*296*-*5p* in PCV2-infected YL pigs than in PCV2-infected LW pigs, we can speculate that *ssc*-*miR*-*296*-*5p* may play some roles in the differential expression of *CXCL13* between LW and YL pigs, although we cannot exclude the possibility that any novel miRNA may also participate in the regulation of *CXCL13*.

Overall, for the first time, we showed an association between the expression of porcine CXCL13 gene and lymphocyte depletion in spleen after PCV2 challenge, and CXCL13 is regulated both at transcriptional and posttranscriptional level. We speculate that the decrease of CXCL13 expression in pigs susceptible to PCV2 infection results in more lymphocyte depletion, and therefore causes more severe lesions in the spleen. The SNP −1014 G > A in the promoter region may affect the transcription of *CXCL13* during PCV2 infection, and is therefore a potential DNA marker for resistance to PCV2 in pigs.

## Additional files

**Additional file 1.** **Correlation statistics of the biological replicates.** T1, T2 and T3: mock-infected YL pigs; T4, T5 and T6: PCV2-infected YL pigs.

**Additional file 2.** **Primers used in this study. **CXCL13-F1 to CXCL13-F6 contained an I site at their 5′-ends, and CXCL13-R contained aXho HindIII site at 5′-end, and 3′-UTR-F and 3′-UTR-R contained an XbaI site at 5′-end (underlined). Product size of CXCL13-F2 to CXCL13-F6 represented the length of amplicon by CXCL13-F2 to CXCL13-F6 and CXCL13-R, respectively.

**Additional file 3.** **Statistics of sequencing reads aligned to the genome.** T1, T2 and T3: mock-infected YL pigs; T4, T5 and T6: PCV2-infected YL pigs.

**Additional file 4.** **Heatmap of DEGs in the spleen between mock-infected (T1, T3) and PCV2-infected (T5, T6) YL pigs.** The columns represent different samples and the rows represent the DEGs. Gene expression levels in the samples are indicated by corresponding colors.

**Additional file 5.** **Detailed information about DEGs.**

**Additional file 6.** **Information about the GO terms related to the 12 DEGs.** ^a^FDR: false discovery rate.

**Additional file 7.** **KEGG pathway analysis of the DEGs.**

## Abbreviations

PCVAD: porcine circovirus-associated disease
PCV2: porcine circovirus type 2
LW pig: Laiwu pig
YL pig: Yorkshire × Landrace pig
DEGs: differentially expressed genes
qPCR: quantitative PCR
GO: gene ontology
RT-qPCR: reverse transcription-quantitative PCR
3′-UTR: 3′-untranslated region
SNP: single nucleotide polymorphism
ssc-miR-296-5p: sus scrofa microRNA-296-5p
LW-i: PCV2-infected LW pig
LW-u: PCV2-uninfected LW pig
YL-i: PCV2-infected YL pig
YL-u: PCV2-uninfected YL pig
RNA-seq: RNA-sequencing
hpi: hours post-infection
PI: propidium iodide
MOI: multiplicity of infection
TCID_50_: 50% tissue culture infective dose
SE: standard error

## References

[1] Tischer I, Gelderblom H, Vettermann W, Koch MA (1982). A very small porcine virus with circular single-stranded DNA. Nature.

[2] Karuppannan AK, Opriessnig T (2017). Porcine circovirus type 2 (PCV2) vaccines in the context of current molecular epidemiology. Viruses.

[3] Segales J (2012). Porcine circovirus type 2 (PCV2) infections: clinical signs, pathology and laboratory diagnosis. Virus Res.

[4] Opriessnig T, Meng XJ, Halbur PG (2007). Porcine circovirus type 2 associated disease: update on current terminology, clinical manifestations, pathogenesis, diagnosis, and intervention strategies. J Vet Diagn Invest.

[5] Alarcon P, Rushton J, Wieland B (2013). Cost of post-weaning multi-systemic wasting syndrome and porcine circovirus type-2 subclinical infection in England—an economic disease model. Prev Vet Med.

[6] Allan GM, Ellis JA (2000). Porcine circoviruses: a review. J Vet Diagn Invest.

[7] Meng XJ (2013). Porcine circovirus type 2 (PCV2): pathogenesis and interaction with the immune system. Annu Rev Anim Biosci.

[8] Fenaux M, Halbur PG, Haqshenas G, Royer R, Thomas P, Nawagitgul P, Gill M, Toth TE, Meng XJ (2002). Cloned genomic DNA of type 2 porcine circovirus is infectious when injected directly into the liver and lymph nodes of pigs: characterization of clinical disease, virus distribution, and pathologic lesions. J Virol.

[9] Lv Y, Dai L, Han H, Zhang S (2012). PCV2 induces apoptosis and modulates calcium homeostasis in piglet lymphocytes in vitro. Res Vet Sci.

[10] Shibahara T, Sato K, Ishikawa Y, Kadota K (2000). Porcine circovirus induces B lymphocyte depletion in pigs with wasting disease syndrome. J Vet Med Sci.

[11] Li Y, Liu H, Wang P, Wang L, Sun Y, Liu G, Zhang P, Kang L, Jiang S, Jiang Y (2016). RNA-seq analysis reveals genes underlying different disease responses to porcine circovirus type 2 in pigs. PLoS ONE.

[12] Lopez-Soria S, Nofrarias M, Calsamiglia M, Espinal A, Valero O, Ramirez-Mendoza H, Minguez A, Serrano JM, Marin O, Callen A, Segales J (2011). Post-weaning multisystemic wasting syndrome (PMWS) clinical expression under field conditions is modulated by the pig genetic background. Vet Microbiol.

[13] Meerts P, Misinzo G, McNeilly F, Nauwynck HJ (2005). Replication kinetics of different porcine circovirus 2 strains in PK-15 cells, fetal cardiomyocytes and macrophages. Arch Virol.

[14] Opriessnig T, Fenaux M, Thomas P, Hoogland MJ, Rothschild MF, Meng XJ, Halbur PG (2006). Evidence of breed-dependent differences in susceptibility to porcine circovirus type-2-associated disease and lesions. Vet Pathol.

[15] Opriessnig T, Patterson AR, Madson DM, Pal N, Rothschild M, Kuhar D, Lunney JK, Juhan NM, Meng XJ, Halbur PG (2009). Difference in severity of porcine circovirus type two-induced pathological lesions between Landrace and Pietrain pigs. J Anim Sci.

[16] Zhang P, Wang L, Li Y, Jiang P, Wang Y, Wang P, Kang L, Wang Y, Sun Y, Jiang Y (2018). Identification and characterization of microRNA in the lung tissue of pigs with different susceptibilities to PCV2 infection. Vet Res.

[17] Gellert A, Salanki K, Tombacz K, Tuboly T, Balazs E (2012). A cucumber mosaic virus based expression system for the production of porcine circovirus specific vaccines. PLoS One.

[18] Trapnell C, Roberts A, Goff L, Pertea G, Kim D, Kelley DR, Pimentel H, Salzberg SL, Rinn JL, Pachter L (2012). Differential gene and transcript expression analysis of RNA-seq experiments with TopHat and Cufflinks. Nat Protoc.

[19] Anders S, Huber W (2010). Differential expression analysis for sequence count data. Genome Biol.

[20] Benjamini Y, Hochberg Y (1995). Controlling the false discovery rate: a practical and powerful approach to multiple testing. J R Statist Soc B.

[21] da Huang W, Sherman BT, Lempicki RA (2009). Bioinformatics enrichment tools: paths toward the comprehensive functional analysis of large gene lists. Nucleic Acids Res.

[22] Kanehisa M, Goto S, Kawashima S, Okuno Y, Hattori M (2004). The KEGG resource for deciphering the genome. Nucleic Acids Res.

[23] Fernandes LT, Tomas A, Bensaid A, Perez-Enciso M, Sibila M, Sanchez A, Segales J (2009). Exploratory study on the transcriptional profile of pigs subclinically infected with porcine circovirus type 2. Anim Biotechnol.

[24] ImageJ. http://imagej.nih.gov/ij/. Accessed 26 June 2012

[25] Chang T, Huang H, Hsu J, Weng S, Horng J, Huang H (2013). An enhanced computational platform for investigating the roles of regulatory RNA and for identifying functional RNA motifs. BMC Bioinform.

[26] Transcriptome analysis of swine spleen after infection with PCV2. https://www.ncbi.nlm.nih.gov/geo/query/acc.cgi?acc=GSE112152. Accessed 10 Feb 2019

[27] Opriessnig T, Janke BH, Halbur PG (2006). Cardiovascular lesions in pigs naturally or experimentally infected with porcine circovirus type 2. J Comp Pathol.

[28] Lin C, Jeng C, Liu J, Lin E, Chang C, Huang Y, Tsai Y, Chia M, Wan C, Pang V (2013). Immune gene expression profiles in swine inguinal lymph nodes with different viral loads of porcine circovirus type 2. Vet Microbiol.

[29] Li W, Liu S, Wang Y, Deng F, Yan W, Yang K, Chen H, He Q, Charreyre C, Audoneet JC (2013). Transcription analysis of the porcine alveolar macrophage response to porcine circovirus type 2. BMC Genomics.

[30] Legler DF, Loetscher M, Roos RS, Clark-Lewis I, Baggiolini M, Moser B (1998). B cell-attracting chemokine 1, a human CXC chemokine expressed in lymphoid tissues, selectively attracts B lymphocytes via BLR1/CXCR5. J Exp Med.

[31] Moser B, Loetscher P (2001). Lymphocyte traffic control by chemokines. Nat Immunol.

[32] Allen CD, Cyster JG (2008). Follicular dendritic cell networks of primary follicles and germinal centers: phenotype and function. Semin Immunol.

[33] Katakai T, Hara T, Sugai M, Gonda H, Shimizu A (2003). Th1-biased tertiary lymphoid tissue supported by CXC chemokine ligand 13-producing stromal network in chronic lesions of autoimmune gastritis. J Immunol.

[34] Ohtani H, Komeno T, Agatsuma Y, Kobayashi M, Noguchi M, Nakamura N (2015). Follicular dendritic cell meshwork in angioimmunoblastic T-cell lymphoma is characterized by accumulation of CXCL13(+) cells. J Clin Exp Hematop.

[35] Allen CD, Okada T, Cyster JG (2007). Germinal-center organization and cellular dynamics. Immunity.

[36] Ansel KM, Ngo VN, Hyman PL, Luther SA, Forster R, Sedgwick JD, Browning JL, Lipp M, Cyster JG (2000). A chemokine-driven positive feedback loop organizes lymphoid follicles. Nature.

[37] Amft N, Curnow SJ, Scheel-Toellner D, Devadas A, Oates J, Crocker J, Hamburger J, Ainsworth J, Mathews J, Salmon M, Bowman SJ, Buckley CD (2001). Ectopic expression of the B cell-attracting chemokine BCA-1 (CXCL13) on endothelial cells and within lymphoid follicles contributes to the establishment of germinal center-like structures in Sjogren’s syndrome. Arthritis Rheum.

[38] Muller G, Hopken UE, Lipp M (2003). The impact of CCR7 and CXCR5 on lymphoid organ development and systemic immunity. Immunol Rev.

[39] Shi K, Hayashida K, Kaneko M, Hashimoto J, Tomita T, Lipsky PE, Yoshikawa H, Ochi T (2001). Lymphoid chemokine B cell-attracting chemokine-1 (CXCL13) is expressed in germinal center of ectopic lymphoid follicles within the synovium of chronic arthritis patients. J Immunol.

[40] Chunsong H, Yuling H, Li W, Jie X, Gang Z, Qiuping Z, Qingping G, Kejian Z, Li Q, Chang AE, Youxin J, Jinquan T (2006). CXC chemokine ligand 13 and CC chemokine ligand 19 cooperatively render resistance to apoptosis in B cell lineage acute and chronic lymphocytic leukemia CD23 + CD5 + B cells. J Immunol.

[41] Smedbakken LM, Halvorsen B, Daissormont I, Ranheim T, Michelsen AE, Skjelland M, Sagen EL, Folkersen L, Krohg-Sorensen K, Russell D, Holm S, Ueland T, Fevang B, Hedin U, Yndestad A, Gullestad L, Hansson GK, Biessen EA, Aukrust P (2012). Increased levels of the homeostatic chemokine CXCL13 in human atherosclerosis—potential role in plaque stabilization. Atherosclerosis.

[42] Ouyang T, Liu XH, Ouyang HS, Ren LZ (2018). Mouse models of porcine circovirus 2 infection. Anim Models Exp Med.

[43] Alberdi M, Iglesias M, Tejedor S, Merino R, Lopez-Rodriguez C, Aramburu J (2017). Context-dependent regulation of Th17-associated genes and IFNgamma expression by the transcription factor NFAT5. Immunol Cell Biol.

[44] Cheung CY, Ko BC (2013). NFAT5 in cellular adaptation to hypertonic stress—regulations and functional significance. J Mol Signal.

[45] Kuper C, Beck FX, Neuhofer W (2015). Generation of a conditional knockout allele for the NFAT5 gene in mice. Front Physiol.

[46] Kasza A (2013). Signal-dependent Elk-1 target genes involved in transcript processing and cell migration. Biochim Biophys Acta.

[47] Odrowaz Z, Sharrocks AD (2012). ELK1 uses different DNA binding modes to regulate functionally distinct classes of target genes. PLoS Genet.

