# Supplementary material for: Suppression of lymphocyte apoptosis in spleen by CXCL13 after porcine circovirus type 2 infection and regulatory mechanism of CXCL13 expression in pigs
Source: Vet Res. 2019 Feb 28;50:17. doi: 10.1186/s13567-019-0634-2 (PMC6394056; doi:10.1186/s13567-019-0634-2)
Supplement: Supplementary file 1 — Additional file 1. Correlation statistics of the biological replicates. T1, T2 and T3: mock-infected YL pigs; T4, T5 and T6: PCV2-infected YL pigs. [file 13567_2019_634_MOESM1_ESM.doc]

**Additional file 1 Correlation statistics of the biological replicates.**

| **Sample 1** | **Sample 2** | **R^2^** |
| --- | --- | --- |
| T1 | T2 | 0.8589 |
| T1 | T3 | 0.9862 |
| T2 | T3 | 0.9029 |
| T4 | T5 | 0.8324 |
| T4 | T6 | 0.8610 |
| T5 | T6 | 0.9706 |

T1, T2 and T3: mock-infected YL pigs; T4, T5 and T6: PCV2-infected YL pigs.
