# Supplementary material for: Suppression of lymphocyte apoptosis in spleen by CXCL13 after porcine circovirus type 2 infection and regulatory mechanism of CXCL13 expression in pigs
Source: Vet Res. 2019 Feb 28;50:17. doi: 10.1186/s13567-019-0634-2 (PMC6394056; doi:10.1186/s13567-019-0634-2)
Supplement: Supplementary file 2 — Additional file 2. Primers used in this study. CXCL13-F1 to CXCL13-F6 contained an I site at their 5′-ends, and CXCL13-R contained aXho HindIII site at 5′-end, and 3′-UTR-F and 3′-UTR-R contained an XbaI site at 5′-end (underlined). Product size of CXCL13-F2 to CXCL13-F6 represented the length of amplicon by CXCL13-F2 to CXCL13-F6 and CXCL13-R, respectively. [file 13567_2019_634_MOESM2_ESM.doc]

**Additional file 2 Primers used in this study.**

| Name | Primer sequences | Product size (bp) | Annealing temperature (°C) |
| --- | --- | --- | --- |
| HPRT1-F | CATTATGCCGAGGATTTGG | 129 | 50 ~ 65 |
| HPRT1-R | CTTGAGCACACAGAGGGCTA |  |  |
| SOCS5-F | ACAAAGACACAGAGTTCACTGG | 139 | 55 |
| SOCS5-R | AGCGACTTCCTACGGTTCT |  |  |
| KLF11-F | GCTGTTGAGGTGCTGGTTT | 237 | 54 |
| KLF11-R | AGTGTCCCTGTTGATGGCT |  |  |
| OAS1-F | ATGTTTCCGAACGCAGGT | 119 | 56 |
| OAS1-R | AGGAGCCACCCTTCACAACT |  |  |
| OAS2-F | CAACTCTGATGGTTCCCTTG | 204 | 56 |
| OAS2-R | ATGCTCTGCTCTTTAGCGA |  |  |
| RSAD2-F | AGAGCCGTTTATCCACGAC | 168 | 56 |
| RSAD2-R | TCACAGGAGATGGCAAGAA |  |  |
| MX2-F | GGAAATACGCAAAGCCCA | 209 | 55 |
| MX2-R | TCTGCTGCTCCTGGATGTA |  |  |
| PTGES3-F | CTGGAGTAAGGAATGTGTCATC | 251 | 56 |
| PTGES3-R | GGCAGTCTGGGAAATGTTC |  |  |
| CYCS-F | GGCTCCTGGATTCTCTTACA | 152 | 54 |
| CYCS-R | CTTCCCTTTCTCCCTTCTTC |  |  |
| HGF-F | AATCCCGATGATGATGCC | 89 | 55 |
| HGF-R | CCTTCACAACGAGAAATAGGG |  |  |
| CXCL13-F | ATCTCTGCTTCTCGTGCTG | 180 | 56 |
| CXCL13-R | ACTTCTCTGGTTGGACATCC |  |  |
| ACTC1-F | CAGAGCAAGCGAGGTATTC | 146 | 53 |
| ACTC1-R | TCAGTGAGCAGAGTAGGGTG |  |  |
| XDH-F | TTTCTGCCAATGCCTGCCT | 215 | 61 |
| XDH-R | AATCTCCTCAACGGTGGGCT |  |  |
| CXCL13-F1 | CCGCTCGAGGAGCACACAATAGCAGTGA | 3486bp | 65 |
| CXCL13-R1 | CCCAAGCTTGCCTCAAGGTGGAGTTCA |  |  |
| CXCL13-F2 | CCGCTCGAGAGACAGAAGAAAGGGCGGT | 2745bp | 65 |
| CXCL13-F3 | CCGCTCGAGTTTCGCTGCTCAGATTGC | 2284bp | 65 |
| CXCL13-F4 | CCGCTCGAGCATCCCATACATACCAGACACT | 1611bp | 65 |
| CXCL13-F5 | CCGCTCGAGTTATCAGGAAGTCATCCCG | 1168bp | 65 |
| CXCL13-F6 | CCGCTCGAGATCTACTTGCCCTCTTCCAG | 668bp | 65 |
| -3065T-F | TGAGCCTGTCATTACTATTGTTTAACAAGTTCGCAGAAGGC | 8283bp | 64 |
| -3065T-R | GCCTTCTGCGAACTTGTTAAACAATAGTAATGACAGGCTCA |  |  |
| -2991G-F | GCTATTTCTACCTTGAAGTGTAGATAGGTGGCTGGTTGCT | 8283bp | 64 |
| -2991G-R | AGCAACCAGCCACCTATCTACACTTCAAGGTAGAAATAGC |  |  |
| -2473G-F | AACCTTAAACCAATTGGTAATGGATGGACGGATGG | 8283bp | 64 |
| -2473G-R | CCATCCGTCCATCCATTACCAATTGGTTTAAGGTT |  |  |
| -1757C-F | GGAGTGCTACACATGAAACAGCAATTGTTTTCATATGGTG | 8283bp | 64 |
| -1757C-R | CACCATATGAAAACAATTGCTGTTTCATGTGTAGCACTCC |  |  |
| -1014G-F | CTCCATACCAGTCCGGAAATTTTTCTTCTGACCTGAGT | 8283bp | 68 |
| -1014G-R | GGTCAGAAGAAAAATTTCCGGACTGGTATGGAGTGAAAAG |  |  |
| -712T-F | CGGATGAAGGTCTAATGACACCTTCCAATGACACC | 8283bp | 64 |
| -712T-R | GGTGTCATTGGAAGGTGTCATTAGACCTTCATCCG |  |  |
| -604C-F | TTCAAGACACAGCCACACTAGGGAGGGGGTATC | 8283bp | 60 |
| -604C-R | GATACCCCCTCCCTAGTGTGGCTGTGTCTTGAA |  |  |
| -474G-F | CAGTAACCTAGTCCGCCCAGGGATCTTGTATATAAGGAGAC | 8283bp | 64 |
| -474G-R | AAGATCCCTGGGCGGACTAGGTTACTGTTTTGCTAC |  |  |
| -1014SNP-F | CATCCCATACATACCAGACACT | 1160 | 54 |
| -1014SNP-R | CTTTGTGTTCTCAGCATCCA |  |  |
| 3'UTR-F | GCTCTAGACGCCAATAACCCCACCAA | 787 | 60 |
| 3'UTR-R | GCTCTAGATTAAGTAAAACAATTGCTCAAGTTT |  |  |
| 3'UTR-mut-F | GAGTATCTGGTGGGGGGAAAAAGCTGGTGTTCAATTCT | 5797 | 55 |
| 3'UTR-mut-R | AGAATTGAACACCAGCTTTTTCCCCCCACCAGATACTC |  |  |
| 5S rRNA | GGTTAGTACTTGGATGGGAGACTGCCT |  | 60 |
| ssc-miR-296-5p | CCCCCCCCAATCCTGTAAA |  | 60 |

CXCL13-F1 to CXCL13-F6 contained an Xho I site at their 5'-ends, and CXCL13-R1 contained a Hind III site at 5'-end, and 3'-UTR-F and 3'-UTR-R contained an Xba I site at 5'-end (underlined). Product size of CXCL13-F2 to CXCL13-F6 represented the length of amplicon by CXCL13-F2 to CXCL13-F6 and CXCL13-R1, respectively.
