# Supplementary material for: Suppression of lymphocyte apoptosis in spleen by CXCL13 after porcine circovirus type 2 infection and regulatory mechanism of CXCL13 expression in pigs
Source: Vet Res. 2019 Feb 28;50:17. doi: 10.1186/s13567-019-0634-2 (PMC6394056; doi:10.1186/s13567-019-0634-2)
Supplement: Supplementary file 3 — Additional file 3. Statistics of sequencing reads aligned to the genome. T1, T2 and T3: mock-infected YL pigs; T4, T5 and T6: PCV2-infected YL pigs. [file 13567_2019_634_MOESM3_ESM.doc]

**Additional file 3**. Statistics of the sequencing reads aligned to genome.

| **Sample ID** | **Total Reads** | **mapped Reads** | **Perfect Map** | **< = 2bp Mismatch** | **Uniq Map** | **Multiple Map** |
| --- | --- | --- | --- | --- | --- | --- |
| T1 | 38884958 (100%) | 29680913 (76.33%) | 15208031 (51.24%) | 2004655 (6.75%) | 28069445 (94.57%) | 1611468 (5.43%) |
| T2 | 33415774 (100%) | 25641513 (76.73%) | 12489644 (48.71%) | 1815716 (7.08%) | 24268899 (94.65%) | 1372614 (5.35%) |
| T3 | 35342492 (100%) | 27008306 (76.42%) | 14040663 (51.99%) | 1760203 (6.52%) | 25525485 (94.51%) | 1482821 (5.49%) |
| T4 | 35721702 (100%) | 27212167 (76.18%) | 13647955 (50.15%) | 1918396 (7.05%) | 25688210 (94.4%) | 1523957 (5.6%) |
| T5 | 32242824 (100%) | 24638990 (76.42%) | 12281002 (49.84%) | 1675764 (6.8%) | 23336353 (94.71%) | 1302637 (5.29%) |
| T6 | 37024608 (100%) | 28143488 (76.01%) | 14132579 (50.22%) | 2052180 (7.29%) | 26640557 (94.66%) | 1502931 (5.34%) |

T1, T2 and T3: mock-infected YL pigs; T4, T5 and T6: PCV2-infected YL pigs.
