# Supplementary figures and images for: Suppression of lymphocyte apoptosis in spleen by CXCL13 after porcine circovirus type 2 infection and regulatory mechanism of CXCL13 expression in pigs
Source: Vet Res. 2019 Feb 28;50:17. doi: 10.1186/s13567-019-0634-2 (PMC6394056; doi:10.1186/s13567-019-0634-2)

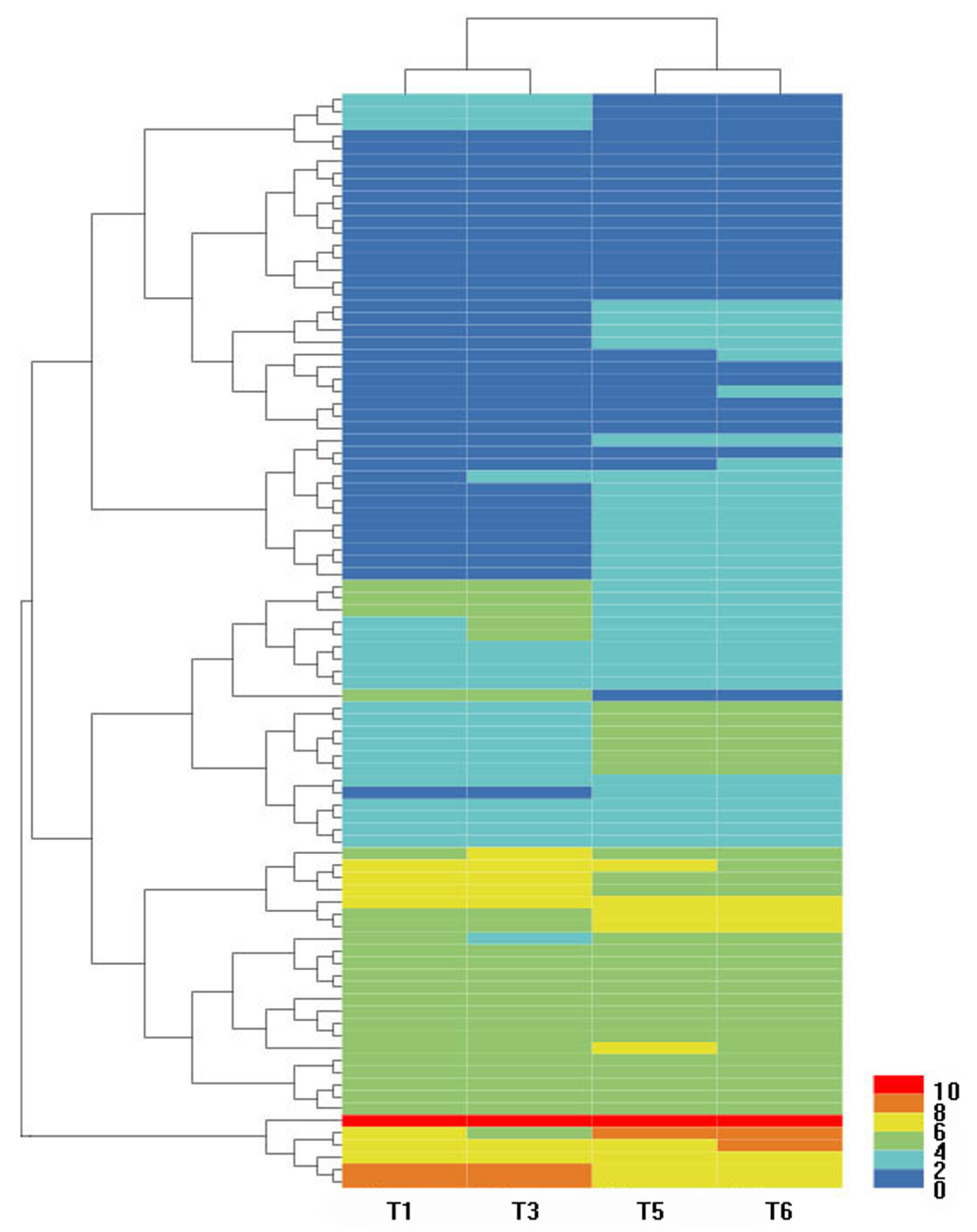

Supplement: Supplementary file 4 — Additional file 4. Heatmap of DEGs in the spleen between mock-infected (T1, T3) and PCV2-infected (T5, T6) YL pigs. The columns represent different samples and the rows represent the DEGs. Gene expression levels in the samples are indicated by corresponding colors. [file 13567_2019_634_MOESM4_ESM.tif]
