# Supplementary material for: Suppression of lymphocyte apoptosis in spleen by CXCL13 after porcine circovirus type 2 infection and regulatory mechanism of CXCL13 expression in pigs
Source: Vet Res. 2019 Feb 28;50:17. doi: 10.1186/s13567-019-0634-2 (PMC6394056; doi:10.1186/s13567-019-0634-2)
Supplement: Supplementary file 6 — Additional file 6. Information about the GO terms related to the 12 DEGs. aFDR: false discovery rate. [file 13567_2019_634_MOESM6_ESM.doc]

**Additional file 6. Information of the GO terms related to the 12** DEGs.

| GO ID | GO term | FDR ^a^ | Genes |
| --- | --- | --- | --- |
| GO:0002230 | positive regulation of defense response to virus by host | 0.34871 | *PDK4* |
| GO:0008285 | negative regulation of cell proliferation | 0.62316 | *KLF11* |
| GO:0008284 | positive regulation of cell proliferation | 0.66386 | *HGF* |
| GO:0008283 | cell proliferation | 0.14300 | *PTGES3, MAP3K11* |
| GO:0001937 | negative regulation of endothelial cell proliferation | 0.16760 | *XDH* |
| GO:0043280 | positive regulation of cysteine-type endopeptidase activity involved in apoptotic process | 0.15392 | *CYCS* |
| GO:0043066 | negative regulation of apoptotic process | 0.68333 | *ACTC1* |
| GO:0006919 | activation of cysteine-type endopeptidase activity involved in apoptotic process | 0.22683 | *XDH* |
| GO:0006915 | apoptotic process | 0.43786 | *CYCS* |
| GO:0043065 | positive regulation of apoptotic process | 0.44772 | *KLF11* |
| GO:1903751 | negative regulation of intrinsic apoptotic signaling pathway in response to hydrogen peroxide | 0.08853 | *HSPH1* |
| GO:0043154 | negative regulation of cysteine-type endopeptidase activity involved in apoptotic process | 0.19609 | *HGF* |
| GO:0006959 | humoral immune response | 0.29987 | *JCHAIN* |
| GO:0002250 | adaptive immune response | 0.29987 | *JCHAIN* |
| GO:0002718 | regulation of cytokine production involved in immune response | 0.08853 | *TRIL* |
| GO:0045087 | innate immune response | 0.05883 | *JCHAIN, RSAD2, OAS1, MX2* |
| GO:0050728 | negative regulation of inflammatory response | 0.08087 | *HGF, SOCS5* |

^a^ FDR: false discovery rate.
